# Identification of MTURN as a trained immunity-related biomarker for heart failure *via* integrative transcriptomic machine learning analysis and experimental validation

**DOI:** 10.3389/fimmu.2026.1739660

**Published:** 2026-02-18

**Authors:** Tianyuan Yang, Zhixin Li, Mingliang Pan, Xiaohong Wang, Wei Huang, Nebahat Ece Kesten, Tianqing Peng, Guo-Chang Fan

**Affiliations:** 1 Department of Pharmacology, Physiology, and Neurobiology, University of Cincinnati College of Medicine, Cincinnati, OH, United States; 2 Division of Pharmaceutical Sciences, James L. Winkle College of Pharmacy, University of Cincinnati, Cincinnati, OH, United States; 3 Department of Critical Care Medicine, Renmin Hospital of Wuhan University, Wuhan, Hubei, China; 4 Division of Cardiovascular Health and Disease, Department of Internal Medicine, University of Cincinnati College of Medicine, Cincinnati, OH, United States; 5 Faculty of Medicine, Ondokuz Mayis University, Atakum, Samsun, Türkiye; 6 London Health Sciences Centre Research Institute, Departments of Medicine and Pathology and Laboratory Medicine, Western University, London, ON, Canada

**Keywords:** biomarker, heart failure, machine learning, MTURN, trained immunity

## Abstract

**Background:**

Heart failure (HF) is a global health burden marked by high morbidity and limited treatment efficacy across subtypes. The lack of reliable molecular biomarkers for heart failure impedes personalized therapy. Emerging evidence suggests that macrophage-trained immunity drives chronic inflammation and cardiac remodeling, highlighting immune-related genes as promising biomarkers.

**Methods:**

We integrated transcriptomic data from five independent HF cohorts and one macrophage-trained immunity model. Differentially expressed genes (DEGs) analysis, weighted gene co-expression network analysis (WGCNA), immune infiltration profiling, and six machine-learning algorithms were applied to screen immune-related candidate genes. Functional relevance was assessed by gene set enrichment analysis (GSEA) and single-cell RNA-seq of human cardiac tissue. Finally, we established a THP-1-derived macrophage trained immunity model to validate the paracrine effects of macrophage Maturin (MTURN) and Piezo-type mechanosensitive ion channel component 1 (PIEZO1) in cardiomyocytes.

**Results:**

Seven hub genes were identified from HF-DEGs, the trained immunity transcriptional signature, and WGCNA co-expression modules. Among them, MTURN, an evolutionarily conserved regulator of differentiation and inflammation, emerged as the most robust candidate, showing consistent upregulation in HF samples across all cohorts with superior diagnostic performance. Importantly, GSEA linked MTURN to innate immune activation and adhesion/signaling pathways. Single-cell RNA-seq analyses of human cardiac tissue revealed MTURN enrichment in cardiac macrophages with a progressive increase along pseudotime. Experimentally, trained immunity macrophages displayed an elevation of glycolytic and inflammatory markers together with increased MTURN and PIEZO1. Accordingly, the conditioned medium collected from such trained macrophages could upregulate expression of HF markers (*i.e*., NPPA/B) in AC16 cardiomyocytes.

**Conclusion:**

Multi-cohort, single-cell RNA-seq, and experimental data collectively suggest MTURN as a trained immunity-related biomarker for the diagnosis of heart failure with a potential link to PIEZO1-mediated cardiac remodeling.

## 1 Introduction

Heart failure (HF) remains a major global health burden, with persistently high incidence and mortality rates. It is one of the leading causes of hospitalization among the elderly and ranks among the costliest chronic diseases worldwide. According to recent estimates, over 60 million people are affected globally, and this number continues to rise (1). Importantly, HF is often accompanied by complications such as arrhythmia, ischemic stroke, and renal failure, which further increase mortality, especially in older adults (2–4). However, despite advances in treatment, HF is a heterogeneous syndrome with significant differences in pathophysiology and drug response among subtypes. As a result, standard therapies such as angiotensin-converting enzyme inhibitors (ACE inhibitors), β-blockers, and angiotensin receptor-neprilysin inhibitors (ARNIs) are effective in only about half of patients. Moreover, the mechanisms underlying various HF subtypes remain poorly understood, limiting the development of broadly effective treatments. Therefore, identifying molecular biomarkers with clear pathophysiological relevance is essential for improving our understanding of HF and advancing precision medicine strategies.

Macrophages, the most abundant immune cell population in the myocardium, serve as critical regulators of chronic inflammation, tissue repair, and fibrosis. In recent years, accumulating evidence has suggested that macrophage immune activity plays a pivotal role in the pathogenesis of HF and its related comorbidities (5). Notably, chronic cardiac stress can induce the activation of “trained immunity” macrophages, particularly the CCR2^+^ subset, which disrupt cardiac immune homeostasis and promote pathological remodeling. These cells may also contribute to increased susceptibility of distal organs (such as skeletal muscle and kidney) to injury and dysfunction (6). In addition, compared to other immune cells, cardiac macrophages exhibit higher and more specific expression of genes associated with inflammation and remodeling; however, their underlying epigenetic characteristics remain largely undefined. It is worth noting that the trained immunity of macrophages has been demonstrated to play a key role in sustaining chronic vascular inflammation and promoting plaque formation in atherosclerosis (7). However, the molecular drivers of this reprogramming remain poorly defined, particularly in the context of macrophage-mediated innate immune memory, or “trained immunity”, which may represent a key axis linking inflammation and cardiac dysfunction. Therefore, exploring novel molecular signals underlying macrophage epigenetic reprogramming in trained immunity may clarify their roles in HF progression.

With the widespread adoption of high-throughput sequencing technologies, the growing availability of public datasets offers valuable opportunities to identify therapeutic targets for various diseases. Here, we integrated HF bulk transcriptomes from multiple independent cohorts with a macrophage trained immunity signature, coupled with weighted gene co-expression network analysis (WGCNA), CIBERSORT, and six complementary machine-learning algorithms to prioritize candidate genes. We then assessed functional relevance by single-gene gene set enrichment analysis (GSEA) and single-cell RNA-seq (scRNA-seq), focusing on cellular specificity and pseudotime dynamics. Finally, we incorporated an *in vitro* trained immunity model using THP-1-derived macrophages to experimentally validate the bioinformatic findings. This integrative strategy aims to identify novel immune-related biomarkers linking macrophage-trained immunity to the pathogenesis of heart failure, providing a foundation for subsequent mechanistic exploration.

## 2 Methods

### 2.1 Dataset collection

We collected five independent HF datasets, including GSE135055, GSE203160, GSE48166, GSE198945, and GSE116250, all containing bulk RNA-seq data from cardiac tissues of human HF patients and healthy controls. In addition, we selected our previously obtained human cardiac bulk RNA-seq datasets GSE165303 for validation. Additionally, GSE235897 was included, providing bulk RNA-seq data of human monocyte-derived macrophages (hMDMs) under trained immunity conditions. All the above datasets were obtained from the GEO public database (https://www.ncbi.nlm.nih.gov/geo/). For single-cell transcriptomic data, we accessed the SCP1303 project from the Broad Institute (https://singlecell.broadinstitute.org/single_cell), which includes raw scRNA-seq data from failing human hearts with dilated and hypertrophic cardiomyopathy. During data preprocessing, probe IDs were mapped to gene symbols. For genes associated with multiple probes, the probe with the highest average expression was retained as the representative. Detailed information on sample composition, disease etiology, sequencing platforms, sample sizes, and direct accession links for each dataset, is provided in the **Supplementary Methods**.

### 2.2 Identification of differentially expressed genes

The Fragments Per Kilobase of transcript per million mapped reads (FPKM) values from the GSE135055 and GSE235897 datasets were log_2_(X + 1) transformed, and all subsequent analyses were performed using R version 4.4.2. Differentially expressed genes (DEGs) between groups were identified using the “limma” package (version 3.62.2), with significance thresholds set at *p* < 0.05 and |log2FC| > 0.585 (corresponding to approximately a 1.5-fold change). The results were visualized using volcano plots and hierarchical clustering heatmaps, which were generated with “ggplot2” package (version 3.5.2) and “pheatmap” package (version 1.0.12).

### 2.3 Functional enrichment analysis

To explore the potential biological functions and pathways implicated by the differentially expressed genes identified from the GSE135055 dataset, Kyoto encyclopedia of genes and genomes (KEGG) and Gene ontology (GO) enrichment analyses were performed on the DEGs using the “clusterProfiler” package (version 4.14.6), based on the “org.Hs.eg.db annotation” package (version 3.20.0), with significance defined as *p* < 0.05. The enrichment results were visualized using the “enrichplot” package (version 1.26.6) and the “ggplot2” package (version 3.5.2). To further evaluate innate immune-related transcriptional alterations at the pathway level, GSEA was performed on the GSE135055 dataset to assess pathway-level alterations between heart failure and healthy control samples. A pre-ranked gene list was generated based on gene expression between the two groups. Gene sets were obtained from the Molecular signatures database (MSigDB), including REACTOME_Innate_Immune_System from the Reactome pathway collection (C2: CP: Reactome) and GOBP_Innate_Immune_Response from the Gene Ontology Biological Process collection (C5: GO: BP). GSEA was conducted using the GSEA (version 4.4.0) following the pre-standard analysis workflow (8). Statistical significance was evaluated using normalized enrichment score (NES), along with nominal p values, false discovery rate (FDR) q values, and family-wise error rate (FWER) p values, as provided by the GSEA software.

### 2.4 Immune infiltration and functional analysis

To characterize the immune cellular landscape and coordinated immune interactions associated with heart failure, we identified immune cell subpopulations by mapping sequencing data to a validated immune cell signature matrix (LM22) representing 22 distinct immune cell types. The CIBERSORT algorithm was employed to quantify the relative abundance of these immune subpopulations and to compare compositional differences among samples (9). The resulting CIBERSORT-derived cell proportion matrix was extracted, converted to numeric format, and immune cell types exhibiting zero variance across samples were excluded to avoid spurious correlations. Spearman correlation coefficients were then calculated across immune cell types to characterize immune cell-cell association patterns. For visualization, the correlation matrix was rendered as an upper-triangular bubble plot (with the diagonal set to 1), with circle size proportional to *p* value and color indicating correlation direction; the correlation matrix was also exported for downstream analyses. To quantify coordinated shifts between immune populations, we additionally computed pairwise immune cell ratios from the CIBERSORT-derived fractions. For each sample and any two immune cell types “A” and “B”, the “A”/”B” ratio was defined as: (f_A + ϵ)/(f_B + ϵ), where f denotes the estimated cell fraction and ϵ = 1 × 10^-6^ is a small pseudocount added to prevent division-by-zero artifacts when a cell type fraction approached zero. For visualization and group comparisons, ratios were log_2_-transformed as log_2_[(f_A + ϵ)/(f_B + ϵ)]. To further explore the correlations among immune functions, we selected ten representative innate immune-related subfunctions. Detailed descriptions of the selected pathways and the gene set-based scoring method are provided in the **Supplementary Methods**. Briefly, a Spearman correlation analysis was conducted to construct an immune functional interaction network, where correlations with a coefficient ≥ 0.6 were considered indicative of strong synergistic interactions. The network was visualized using Cytoscape (version 3.10.1), with node degree values used to highlight key functional interactions.

### 2.5 Weighted gene co-expression network analysis

To identify core gene modules and key genes driving the transcriptional alterations observed in the GSE135055 dataset, WGCNA was performed using the “WGCNA” package (version 1.72-5) to identify gene co-expression modules associated with clinical traits. Genes were filtered based on expression variance, retaining the top 25% of the most variable genes. A soft-thresholding power (β) was selected to approximate scale-free topology (R² ≥ 0.90), and the resulting adjacency matrix was transformed into a topological overlap matrix (TOM) to measure network connectivity. Modules were identified via hierarchical clustering with dynamic tree cutting (minimum module size = 30), and similar modules were merged using a cut height of 0.25. Pearson correlation analysis was used to assess relationships between module eigengenes and clinical traits, and the two modules showing the strongest associations were selected for further analysis. All network construction and visualization were performed in R, with additional support from the “dplyr” package (version 1.1.4).

### 2.6 Machine learning for gene selection

To determine which candidate gene plays more prominent and influential roles in heart failure, potential candidate genes were first screened by integrative analysis of heart failure-associated differentially expressed genes, high-weight genes derived from WGCNA, and trained immunity-related genes using a Venn diagram-based approach. A machine learning-based prioritization framework was then established in R to systematically rank these candidates together with identification of the core genes. The pre-selected genes from GSE135055 were used as input features, and gene expression data were merged with phenotypic group labels to construct a classification dataset. Stratified random sampling was applied to divide the samples into a training set (70%) and a test set (30%). Prior to model training, all gene expression features were centered and scaled based on the training data to ensure comparability across features. Six widely used machine learning models were trained using the “caret” package (version 7.0-1), including random forest (RF), support vector machine with a radial basis kernel (SVM), logistic regression (GLM), least absolute shrinkage and selection operator (LASSO) regression, k-nearest neighbors (KNN), and neural networks (NNET), with five-fold cross-validation employed to evaluate model performance. Model performance was initially evaluated using cross-validation-based classification metrics, and model generalizability was further assessed on the independent test set using the “DALEX” package (version 2.4.3). Predicted probabilities for the positive class were used to calculate probability residuals and root mean square error (RMSE) to characterize prediction uncertainty and calibration. Partial dependence plots were generated to visualize the marginal effect of individual genes on model predictions, while reverse cumulative distribution plots and boxplots of residuals were used to evaluate predictive accuracy. In addition, permutation-based variable importance analysis was performed to quantify and visualize the contribution of each gene across different models. To further screen candidate genes, we incorporated the mean variable-importance of different genes derived from machine learning models into principal component analysis (PCA). Specifically, for each candidate gene, a variable-importance matrix obtained from six models (RF, SVM, GLM, LASSO, KNN, and NNET). The importance values were z-score standardized across genes within each model (centered and scaled), and subsequently re-weighted to integrate model contributions based on their relative ranking derived from the preceding machine learning evaluation. A 3D PCA plot was generated based on these values, and genes that are farthest along each of the first three principal components (PC1, PC2, and PC3) were selected as potential validation targets. All figures were generated and exported using the “ggplot2” and “fs” packages (version 1.6.6).

### 2.7 Integrated biomarker evaluation

To evaluate the classification performance and cross-dataset robustness of the selected genes, receiver operating characteristic (ROC) analysis was performed using the “pROC” package (version 1.18.5). ROC curves were generated in 4 independent heart failure cohorts for each gene, including GSE203160, GSE116250, GSE198945, and GSE48166. For each dataset, a gene-signature score was calculated by aggregating the expression values of the selected genes, and ROC curves were constructed based on the signature scores and corresponding group labels. The area under the curve (AUC) was calculated for each dataset and visualized in a combined ROC plot. Additionally, meta-analysis was performed using the “meta” package (version 8.1-0) to synthesize gene level standardized mean differences (SMDs) across independent datasets. Forest plots were generated to visualize dataset-specific and pooled effect sizes. Combined effect estimates from random-effects models, together with heterogeneity statistics, were used to assess the consistency and stability of gene expression patterns across datasets.

### 2.8 Single-gene gene set enrichment analysis

Single-gene GSEA was performed using a single-gene-based, pre-ranked approach to characterize biological pathways associated with the expression level of the gene of interest. Briefly, genes were ranked according to their Spearman correlation coefficients with the target gene, calculated across samples, and ranked in descending order. To avoid undefined values, minimal expression values were truncated prior to log_2_ transformation. The resulting ranked gene list was used as input for single-gene GSEA analysis. GO gene sets were obtained from the Molecular signatures database (MSigDB) collection (c5.go.v2024.1.Hs.symbols.gmt) and analyzed using the “clusterProfiler” package (version 4.14.6). All GO terms were initially retained (pvalueCutoff = 1), and significantly enriched terms were defined based on a false discovery rate (FDR) q value < 0.25. For visualization, both enrichment curves and ridge plots were generated using the “enrichplot” package (version 1.26.6) to highlight the top enriched GO terms.

### 2.9 Single-cell RNA-seq data analysis

For single-cell transcriptomic analyses, we accessed the SCP1303 project from the Broad Institute Single Cell Portal. which contains raw scRNA-seq data derived from failing human hearts diagnosed with dilated cardiomyopathy (DCM) and hypertrophic cardiomyopathy (HCM), along with non-failing donor controls. The dataset includes gene expression profiles generated using droplet-based scRNA-seq technology, with detailed cell-type annotations provided by the original study. Raw scRNA-seq data were processed in Python (Scanpy version 1.9.3). Cells with low quality were excluded based on standard quality control criteria, including abnormal total UMI counts, gene detection rates, and elevated mitochondrial gene expression. Gene expression matrices were normalized and log-transformed where appropriate, and highly variable genes were identified for downstream dimensionality reduction. PCA was performed, followed by construction of a neighborhood graph and nonlinear dimensionality reduction using Uniform manifold approximation and projection (UMAP) for visualization. Based on curated cell-type annotations from the original dataset, macrophage populations were extracted for focused downstream analyses. To improve computational efficiency and ensure compatibility with trajectory inference, the data were further streamlined by retaining essential expression layers and metadata, generating an intermediate AnnData object for subsequent analysis. The data were then converted in R using zellkonverter (version 1.10.0) into a CellDataSet (CDS) compatible with monocle3 (version 1.3.1). Trajectory inference was conducted using the standard monocle3 workflow, including preprocessing, dimensionality reduction, cell clustering, principal graph learning, and pseudotime ordering. The root cell was manually specified to represent macrophages at the inferred early stage of the trajectory. To characterize transcriptional dynamics along pseudotime, MTURN expression was visualized across the inferred trajectory. Cells were subsequently stratified based on MTURN expression levels, with the top 40% classified as MTURN-high and the bottom 40% as MTURN-low, while intermediate cells were excluded to enhance group separation. Differential gene expression analyses were performed between MTURN-high and MTURN-low macrophages separately within DCM and HCM samples, enabling disease-specific characterization of MTURN-associated transcriptional programs.

### 2.10 Correlation analysis

Pairwise correlations were computed to assess associations between MTURN expression and heart-failure marker genes using normalized bulk RNA-seq expression matrices derived from human cardiac tissue samples in our previous dataset (GSE165303). Expression tables were processed in R using the “data.table” package (version 1.17.8), and correlation analyses were performed across all samples. Two-sided Spearman correlation coefficients and corresponding nominal p values were calculated for each marker, with *p* < 0.05 considered statistically significant. Results were visualized using scatter plots generated with the “ggplot2” package.

### 2.11 *In vitro* experiments

Based on published methods (10–12), we established a macrophage trained immunity model using THP-1-derived macrophages, complementing the Al(OH)_3_/PHAD/mannan signature used in the transcriptomic discovery with an independent β-glucan-based approach. Briefly, THP-1 cells were differentiated into macrophages with phorbol 12-myristate 13-acetate (PMA; 100 nM, 24h), then “trained” with β-glucan (5 µg/mL, 24h). After a 24h rest in complete medium, cells were restimulated with lipopolysaccharide (LPS; 10 ng/mL, 24h); the resulting supernatant was collected as conditioned medium (CM) and applied to AC16 human cardiomyocytes for 24h. Controls used CM was generated from non-trained THP-1-derived macrophages that were restimulated with LPS (10 ng/mL, 24h) and applied to AC16 cells for 24h. THP-1 cells were maintained in RPMI-1640 supplemented with 10% FBS, 1% penicillin-streptomycin (P/S), and 0.05 mM β-mercaptoethanol; THP-1-derived macrophages were cultured in DMEM with 10% FBS and 1% P/S; and AC16 cells were maintained in DMEM/F12 (1:1) supplemented with 10% FBS and 1% P/S. All cells were cultured at 37 °C in a humidified atmosphere containing 5% CO_2_.

### 2.12 RT-qPCR

Total RNA was extracted from THP-1-derived macrophages and AC16 cardiomyocytes using the RNeasy Kit (QIAGEN, #217004) in accordance with the manufacturer’s instructions. First-strand cDNA was generated using SuperScript II Reverse Transcriptase (Invitrogen, #18080044). Then qPCR was performed with AZURE CIELO3 system using SYBR Green Master Mix (Alkali Scientific, #QS1001). The mRNA expression levels were normalized to GAPDH and determined using the 2^-ΔΔCt^ method. All primer sequences for RT-qPCR are listed in **Supplementary Table 1**.

### 2.13 Statistics

All statistical analyses were conducted using GraphPad Prism (version 8.02). Results are expressed as the mean ± standard deviation (SD). Comparisons between two groups were performed using a two-tailed unpaired Student’s T-test. For comparisons involving multiple groups, one-way analysis of variance (ANOVA) followed by Tukey’s *post hoc* test was applied. A p-value less than 0.05 was considered statistically significant (* denotes *p* < 0.05; ** denotes *p* < 0.01).

## 3 Results

### 3.1 Enrichment analysis of DEGs in heart failure cardiac tissue

**Figure 1:**
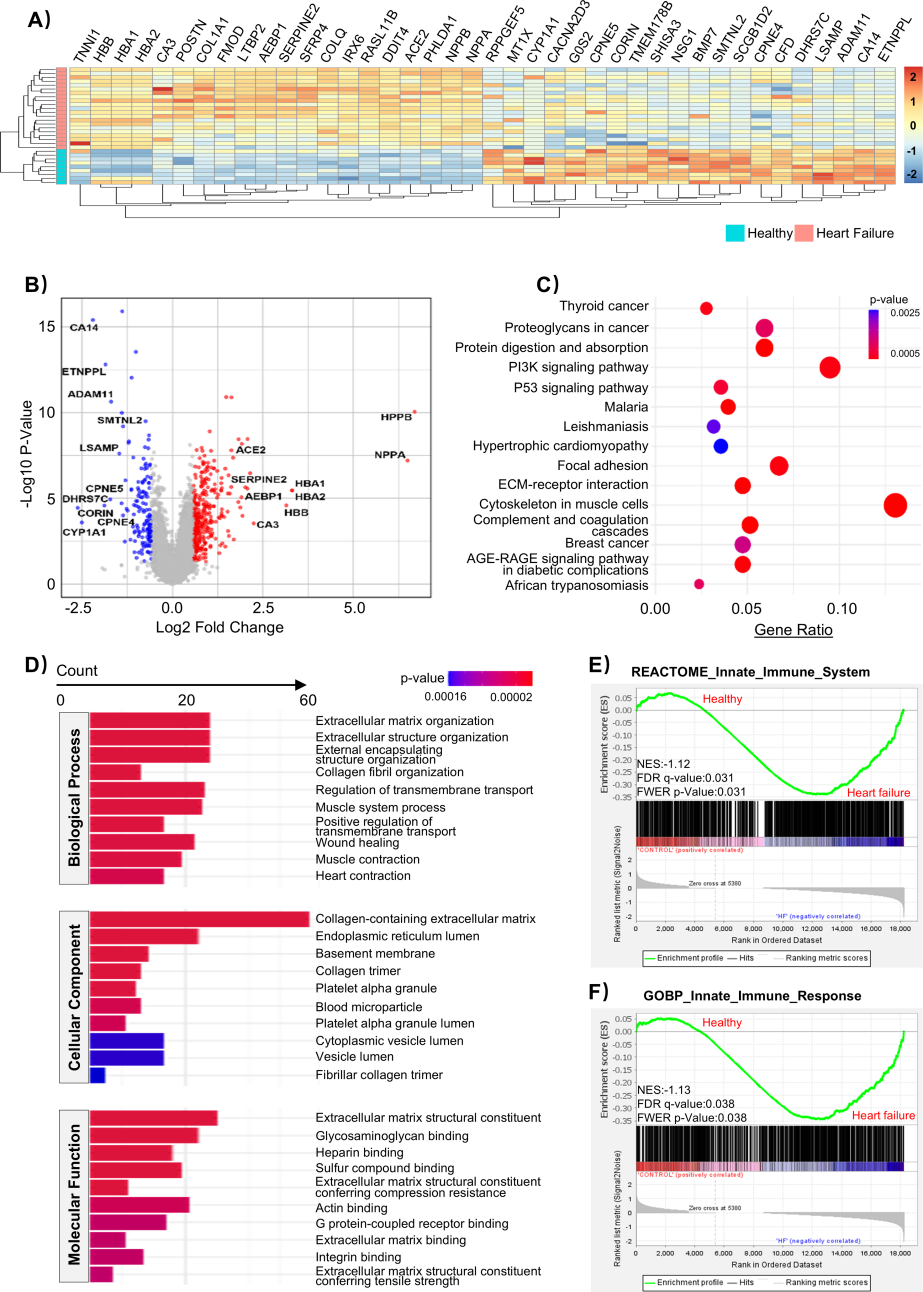
Differential gene expression landscape in left ventricular tissue from the GSE135055 cohort. **(A)** Heatmap showing differentially expressed genes (DEGs) between healthy controls (n = 9) and heart failure patients (n = 21). **(B)** Volcano plot of DEGs (|log_2_FC| ≥ 0.585, *p* < 0.05), identifying 336 upregulated and 146 downregulated genes in the heart failure group relative to controls. **(C)** KEGG enrichment analysis of DEGs (*p* < 0.05). Dot size indicates the number of genes, and dot color denotes the *p* value (blue, higher; red, lower). **(D)** GO enrichment analysis of DEGs across Biological Process (BP), Cellular Component (CC), and Molecular Function (MF). Bar height indicates the number of genes, and bar color denotes the *p* value (blue, higher; red, lower) (*p* < 0.05). **(E, F)** GSEA plots of innate immune-related gene sets in GSE135055 (heart failure *vs.* healthy controls). **(E)** REACTOME Innate Immune System (NES = -1.12, FDR q = 0.031, FWER p = 0.031) and **(F)** GOBP Innate Immune Response (NES = -1.13, FDR q = 0.038, FWER p = 0.038); negative NES indicates enrichment toward the heart failure phenotype based on the ranking direction.

To investigate the transcriptomic alterations associated with heart failure, we first analyzed bulk RNA-seq data from the GSE135055 dataset, including 21 failing hearts and 9 healthy heart tissue samples. Compared with healthy controls, 336 genes were significantly upregulated in the cardiac tissue of heart failure patients, including NPPA and NPPB, which encode atrial and B-type natriuretic peptides and are well-established markers of cardiac stress and heart failure severity (13), as well as HBA1, HBA2, and HBB, which encode hemoglobin subunits and may reflect hypoxia-related or erythrocyte-associated transcriptional alterations in failing hearts (14). Conversely, 146 genes were significantly downregulated, including CA14, which encodes carbonic anhydrase 14 involved in pH regulation (15); ADAM11, a member of the disintegrin and metalloproteinase family implicated in cell-cell interactions (16); CPNE5, a calcium-dependent phospholipid-binding protein that we have previously shown to play a critical role in maintaining aortic integrity during early sepsis (17); and LSAMP, a cell-adhesion molecule linked to tissue structural maintenance (18). Collectively, these suppressed transcripts consistently point to a coordinated attenuation of signaling and structural integrity programs in failing myocardium. (**Figures 1A, B**, **Supplementary Table 2**). To investigate the biological relevance of these DEGs, we performed functional enrichment analyses. KEGG pathway analysis revealed significantly enriched pathways, among which cytoskeleton in muscle cells and the PI3K signaling pathway ranked among the top hits (**Figure 1C**). These genes can be broadly categorized into heart failure-associated stress and remodeling markers (ANKRD1 and FHL1) (19, 20), core sarcomeric and contractile components (MYOM1, MYL3, TNNI1, and TPM3) (21–23), cytoskeleton remodeling and mechanotransduction regulators (XIRP1, XIRP2, and DIAPH1) (24, 25), metabolic enzymes (CKM and ENO2) (26), and extracellular matrix-related genes with immune-modulatory potential (BGN, VCAN, FN1, THBS1, and multiple collagen family members) (27, 28). Such an enrichment pattern is reasonable, as the GSE135055 dataset contains a substantial number of heart failure-associated structural and contractile genes that are well represented in pathways related to muscle cytoskeleton organization and PI3K signaling. To identify immune-specific transcriptional alterations beyond tissue structure-dominated pathways, we next focused on three innate immune-related pathways including malaria, leishmaniasis, and the complement/coagulation cascades. Our analysis showed that these three pathways were enriched for genes involved in complement activation (C3, CFD, CFH/CFHR1, and MASP1) (29), coagulation-inflammation crosstalk (F2R/F2RL2, THBD, PROS1, SERPINE1/2, and VTN) (30), Fc receptor-mediated immune responses (FCGR3A and FCGR3B) (31), as well as endothelial adhesion molecules and key signaling regulators such as VCAM1, JAK2, NFKBIA, and TGFB2 (32, 33). Collectively, these analysis results suggest the presence of a pronounced innate immune activation signature in heart failure. Interestingly, GO enrichment analysis further identified pathways related to tissue remodeling and repair, including collagen fibril organization, collagen-containing extracellular matrix, as well as extracellular matrix structural constituent (**Figure 1D**). Such pathways likely reflect the complex structural and signaling adaptations occurring in the failing myocardium and provide a contextual framework within which immune-related processes may operate. Furthermore, we performed targeted gene set enrichment analysis using two innate immune-related gene sets: REACTOME Innate Immune System (NES = -1.12, FDR q-value = 0.031, and FWER p-Value = 0.031) and GOBP Innate Immune Response (NES = -1.13, FDR q-value = 0.038, and FWER p-Value = 0.038) (**Figures 1E, F**). Both gene sets were significantly enriched in heart failure samples. Together, these findings indicate that dysregulation of innate immune-related functions may contribute to the pathogenesis and progression of heart failure.

### 3.2 Macrophage immune training contributes to heart failure progression

**Figure 2:**
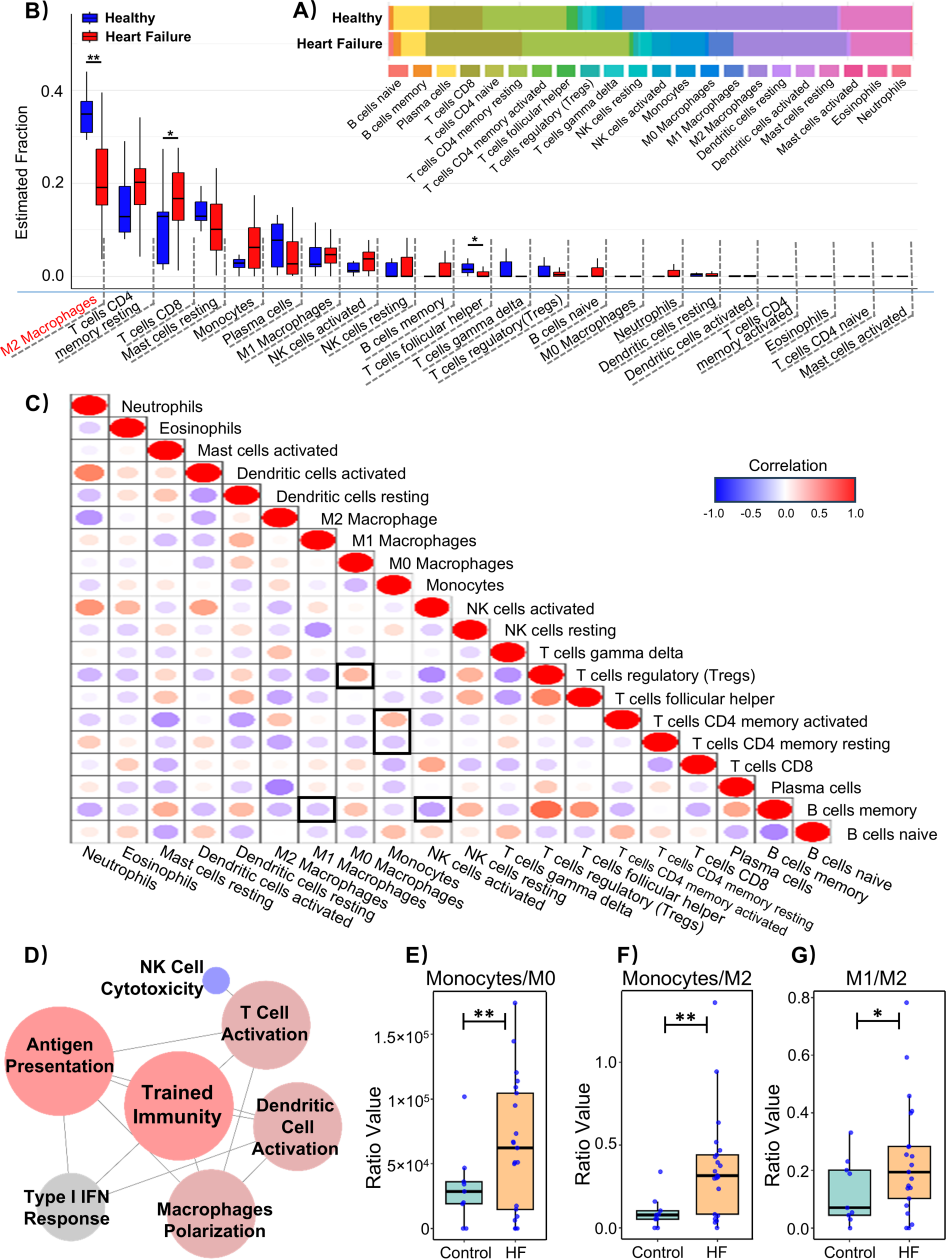
Immune infiltration and functional correlation analysis of the dataset GSE135055 from human failing hearts and healthy controls. **(A)** Proportions of 22 immune cell types in the Heart failure group (n=21) and Healthy group (n=9) estimated by CIBERSORT based on immune cell-specific marker genes. **(B)** Immune cell infiltration analysis (n = 9 for Healthy group, n = 21 for Heart failure group; *p < 0.05, **p < 0.01). **(C)** Spearman correlation matrix of immune cell fractions within the HF group; correlation coefficients range from -1 to 1, and dot color denotes the correlation coefficient. **(D)** Innate immune subfunction functional interaction network constructed from immune infiltration-derived functional scores in GSE135055. Analysis of the **(E)** monocyte-to-M2 macrophage ratio, **(F)** monocyte-to-M0 macrophage ratio, and **(G)** M1-to-M2 macrophage ratio (n = 9 for Healthy group, n = 21 for Heart failure group; *p < 0.05, **p < 0.01).

Next, to investigate the relationship between HF-associated transcriptional alterations and innate immune activation, we quantified immune cell infiltration in the left ventricular cohort (GSE135055) (**Figure 2A**), and found that HF samples exhibited a distinct immune remodeling compared with healthy controls (**Figure 2B**). Specifically, the proportions of M2 macrophages and follicular helper T cells were markedly decreased in HF, whereas CD8^+^ T cells were significantly increased. Of note, a similar reduction in M2-like macrophage signatures has been reported in several independent transcriptomic analyses of failing human myocardium (34–37). Importantly, the deconvolution-inferred “M0/M1/M2” macrophage fractions were not interpreted as definitive evidence of canonical polarization programs, but could be as phenotype-associated transcriptional signatures reflecting immune reprogramming at the tissue level. To figure out the potential relationship underlying these changes across innate immune subfunctions, we constructed an immune functional interaction network using the immune infiltration profiles from GSE135055, and revealed that there was a high interconnectivity between trained immunity- and macrophage polarization-related modules (**Figure 2D**), suggesting that these processes may represent central axes of immunological remodeling in HF. In addition, we examined immune cell-cell correlation patterns within HF samples, which revealed coordinated shifts across myeloid and lymphoid compartments rather than isolated changes (**Figure 2C**). In particular, monocytes were positively correlated with activated CD4^+^ memory T cells, and regulatory T cells (Tregs) were positively associated with M0 macrophages signatures. In contrast, Tregs showed a negative correlation with activated NK cells, and M1 macrophages were inversely correlated with memory B cells. It is important to note here, M0 macrophages in deconvolution frameworks represent transcriptionally inferred, relatively non-polarized myeloid states rather than functionally inert cells; therefore, these correlations could be interpreted as reflecting immune microenvironmental balance and myeloid differentiation potential, rather than direct cell-cell interactions (38, 39). Within this framework, the positive association between Tregs and M0-like macrophage signatures may indicate a relatively immunoregulatory milieu in which macrophage polarization is restrained or delayed, consistent with a transitional myeloid reprogramming state. Accordingly, such coordinated immune remodeling is compatible with sustained innate immune activation and reprogramming, a hallmark of trained immunity described in chronic inflammatory settings (7). Together, these results suggest the presence of trained immunity-like immune reprogramming in HF. Moreover, analysis of immune cell ratios revealed that heart failure patients had significantly elevated ratios of monocytes to M0 and M2 macrophages, along with an increased ratio of M1/M2 compared to healthy controls. (**Figures 2E-G**). These ratio shifts could be interpreted as functional readouts, consistent with trained immunity-associated macrophage reprogramming rather than macrophage polarization. Therefore, dynamic remodeling of macrophage-associated signatures and trained immunity-related immune reprogramming may contribute to HF development and progression.

### 3.3 Identification of potential functional genes related to macrophage trained immunity in heart failure cohorts

**Figure 3:**
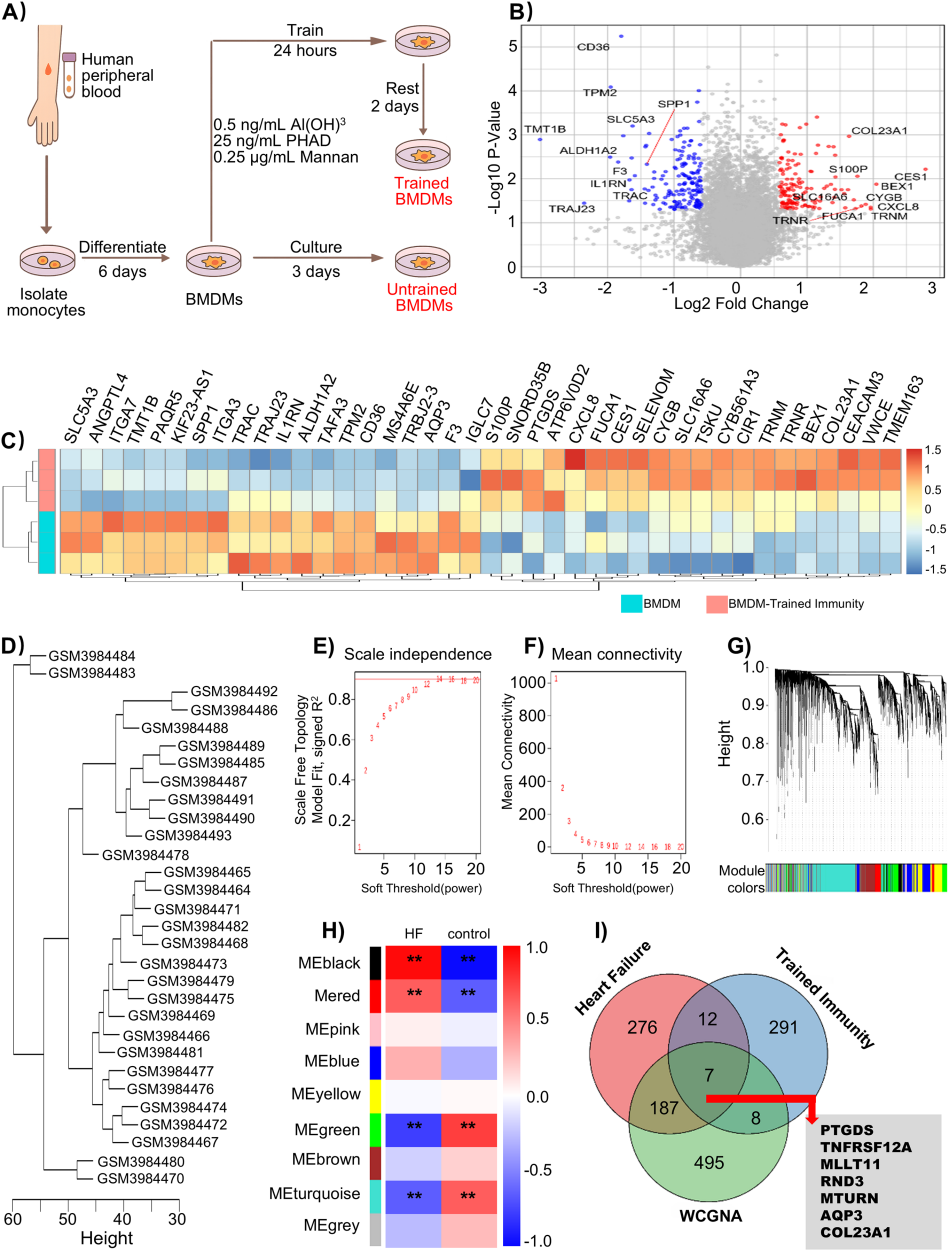
Identification of core genes associated with macrophage immune training and heart failure. **(A)** Schematic overview of human-derived macrophage trained immunity model and transcriptomic profiling workflow (GSE235897). **(B)** The volcano plot and **(C)** DEGs heatmap of hMDMs from trained (n=3) and untrained (n=3) samples in the macrophage-trained immunity dataset GSE235897 (|log2FC| ≥ 0.585, *p* < 0.05). **(D)** Sample clustering dendrogram of GSE135055 dataset based on gene expression profiles. **(E)** Scale-free topology fit index and **(F)** mean connectivity analysis across a range of soft-thresholding powers. **(G)** Cluster dendrogram of genes showing co-expression modules identified by WGCNA in database GSE135055. **(H)** Module-trait heatmap values represent correlation coefficients between healthy controls and HF samples (*p < 0.05, **p < 0.01). **(I)** Venn diagram showing the overlap among heart failure DEGs, trained-immunity DEGs, and WGCNA module genes.

At present, comprehensive studies summarizing gene expression changes associated with trained immunity, particularly in macrophages, are still lacking. Hence, we selected GSE235897 database in which human monocytes were differentiated into macrophages and subsequently trained with a combination of Al(OH)_3_, PHAD, and mannan (**Figure 3A**), as this model provides a basis for investigating the transcriptional changes associated with macrophage-trained immunity. With differential expression analysis, we identified 147 significantly upregulated and 171 significantly downregulated genes in macrophages following training (**Figures 3B, C**, **Supplementary Table 3**). These DEGs could be compiled into a gene set representing the transcriptional signature of trained immunity.

Given that the DEGs derived from the heart-failure dataset (GSE135055) were extensive, we next applied WGCNA to identify the core genes associated with the HF phenotype. After confirming the absence of outlier samples through hierarchical clustering, we selected a soft-thresholding power of 9 to satisfy the scale-free topology criterion (**Figures 3D-G**). Subsequently, multiple gene modules were identified using dynamic tree cutting. Among them, the black module (MEblack, r = 0.92, *p* < 1e-12) was positively correlated with heart failure, while the green module (MEgreen, r = - 0.74, *p* < 1e-6) was negatively correlated. Together, these two modules encompassed a total of 697 genes (**Figure 3H**, **Supplementary Table 4**). Finally, Venn integration of WGCNA module genes, HF DEGs, and the trained-immunity signature identified seven candidate hub genes—PTGDS, TNFRSF12A, MLLT11, RND3, MTURN, AQP3, and COL23A1—that may represent key molecular links between macrophage trained immunity and HF pathogenesis (**Figure 3I**).

### 3.4 Evaluation of diagnostic potential of candidate genes by machine learning

**Figure 4:**
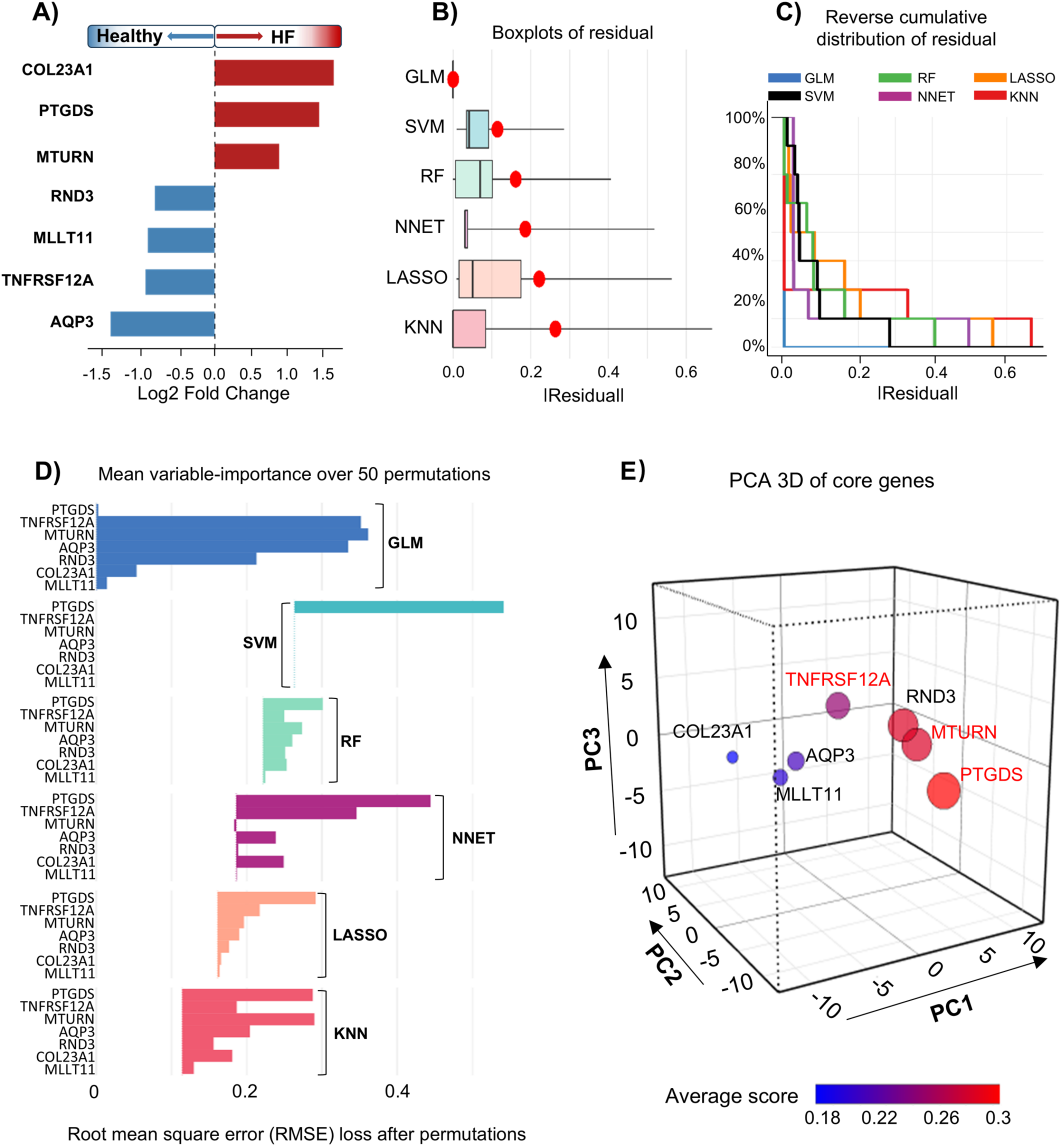
Machine learning-based identification and visualization of core genes associated with heart failure. All analyses were performed using the GSE135055 dataset. **(A)** Log2FC of seven core genes (HF group *vs*. Healthy group). **(B)** Boxplots of absolute probability residuals obtained from six machine learning models. **(C)** Reverse cumulative distribution of absolute residuals from six machine learning models. **(D)** Mean permutations-based variable importance scores calculated across six machine learning models, averaged over 50 permutations. **(E)** 3D PCA of candidate genes based on mean permutation-based variable importance scores, with gene color representing average model score across machine learning models.

Building on the integrative identification of seven candidate genes with distinct expression patterns in heart failure, we next applied multiple machine learning algorithms to assess their diagnostic significance (**Figure 4A**). Specifically, generalized linear model (GLM), support vector machine (SVM), random forest (RF), and neural network-based models were implemented in parallel to capture complementary predictive characteristics across algorithms. Residual boxplots showed that GLM and SVM had more tightly clustered error distributions, with reduced variance and fewer extreme residuals, indicating greater model stability (**Figure 4B**). Meanwhile, reverse cumulative distribution plots of residuals revealed that GLM and NNET yielded the lowest prediction errors across the majority of samples, suggesting superior predictive performance (**Figure 4C**). Furthermore, feature importance rankings further demonstrated that GLM, SVM, and RF maintained stable performance under variable perturbations, indicating enhanced model robustness and reduced sensitivity to noise or sampling variation (**Figure 4D**). To more accurately identify top-performing genes, we conducted a weighted PCA integrating outputs from multiple models to minimize bias introduced by any single algorithm (**Figure 4E**). The PCA results revealed substantial variation in the spatial distribution of genes, with PTGDS, MTURN, and TNFRSF12A located furthest from the centroid in three-dimensional space, reflecting strong and consistent contributions across models. Therefore, these genes exhibit strong discriminative capacity, with MTURN in particular highlighting a close association with macrophage-mediated trained immunity-related transcriptional programs in heart failure.

### 3.5 Cross-cohort validation reveals MTURN as a stable biomarker in heart failure

**Figure 5:**
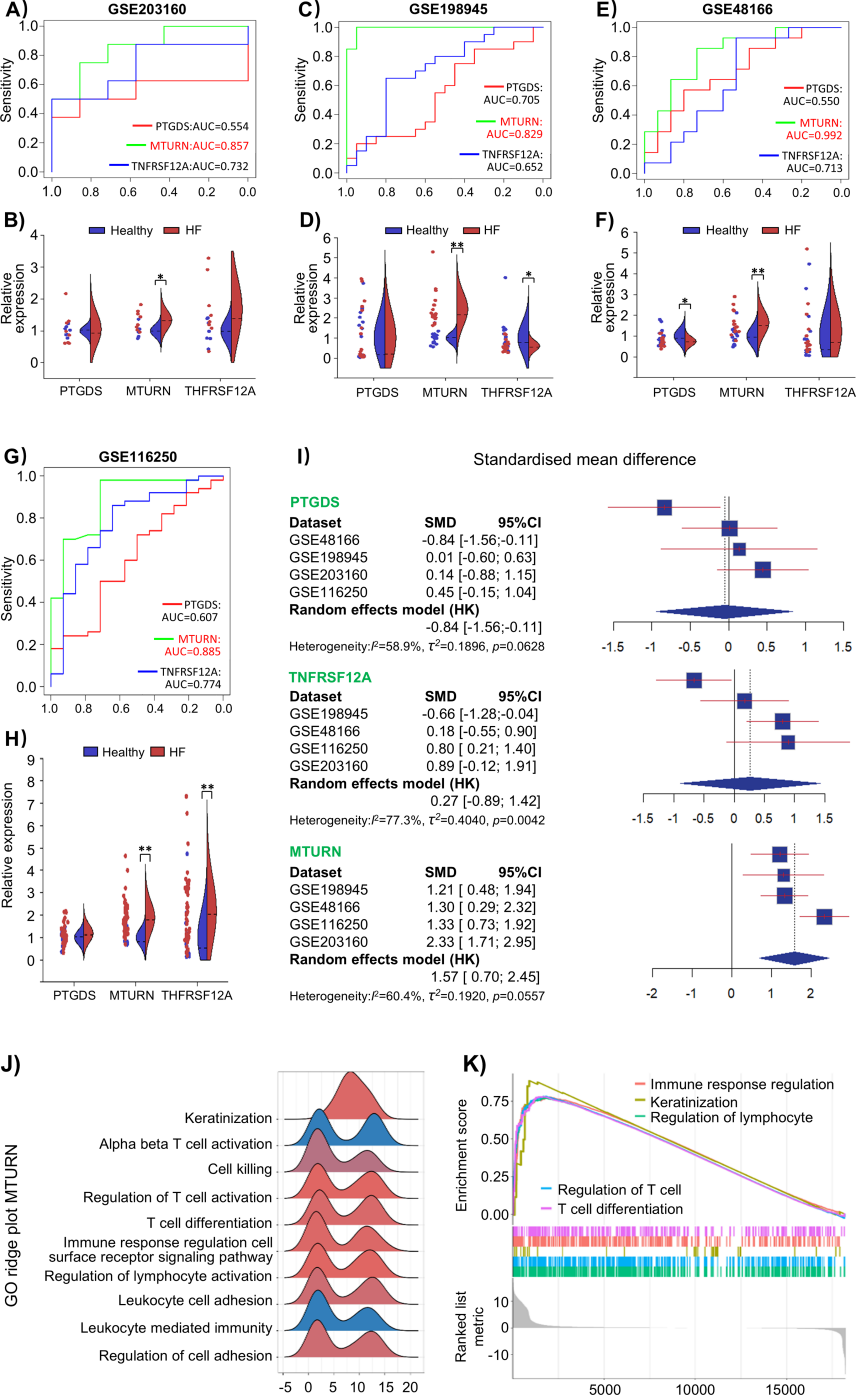
Validation of MTURN in multiple independent heart failure datasets and functional enrichment analysis. **(A)** ROC curve validation and **(B)** gene expression analysis in the GSE203160 dataset. (n = 7 for Healthy group, n = 8 for Heart failure group; *p < 0.05). **(C)** ROC curve validation and **(D)** gene expression analysis in the GSE198945 dataset. (n = 20 for Healthy group, n = 20 for Heart failure group; *p < 0.05, **p < 0.01). **(E)** ROC curve validation and **(F)** gene expression analysis in the GSE48166 dataset. (n = 15 for Healthy group, n = 14 for Heart failure group; *p < 0.05, **p < 0.01). **(G)** ROC curve validation and **(H)** gene expression analysis in the GSE116250 dataset. (n = 14 for Healthy group, n = 50 for Heart failure group; *p < 0.05, **p < 0.01). **(I)** Forest plots from random-effects meta-analysis showing SMDs for PTGDS, TNFRSF12A, and MTURN across independent heart failure cohorts. **(J, K)** single-gene GSEA of GO biological processes based on a pre-ranked gene list derived from heart failure versus control samples, visualized by ridge plot **(J)** and enrichment curve **(K)**, highlighting immune-related pathways associated with MTURN.

To validate the machine learning-derived performance of the candidate genes, especially MTURN, we evaluated their expression and diagnostic value in four independent HF datasets. Importantly, ROC curve analysis demonstrated that MTURN consistently achieved high AUC values across all datasets (maximum AUC = 0.992, outperforming PTGDS and TNFRSF12A (**Figures 5A-C, G**). Furthermore, after normalizing expression data, we assessed the differential expression patterns of these genes and found that MTURN remained consistently upregulated in HF samples (**Figures 5D-F, H**). A subsequent meta-analysis of the combined datasets revealed that MTURN had the highest SMD of 1.57 (95% CI: 0.70-2.45), indicating robust and consistent upregulation in HF samples (**Figure 5I**). In addition, single-gene GSEA revealed that MTURN-associated genes were significantly enriched in immune activation pathways, including leukocyte-mediated immunity, regulation of adhesion molecules, and immune response remodeling (**Figures 5J, K**). Next, to explore the relevance of the trained immunity transcriptional signature to heart failure, we performed correlation analysis between MTURN expression, and the dysregulated genes identified in the trained immunity model. Notably, MTURN showed strong positive correlations with multiple pro-inflammatory and immune activation-related genes (**Supplementary Table 5**), including CXCL8, CEACAM3, CYGB, and COL23A1 (40–42). In contrast, MTURN expression was negatively correlated with genes involved in immune regulation and metabolic homeostasis, such as IL1RN, CD36, and ALDH1A2 (43, 44) (**Supplementary Figure S1**). Collectively, these findings suggest that the elevated expression of MTURN in HF is a consistent and widespread phenomenon, highlighting its strong association with macrophage-mediated immune regulation and underscoring its potential as a promising molecular target for future investigation.

### 3.6 MTURN shows pseudotime-dependent activation and correlates with immune-linked gene upregulation

**Figure 6:**
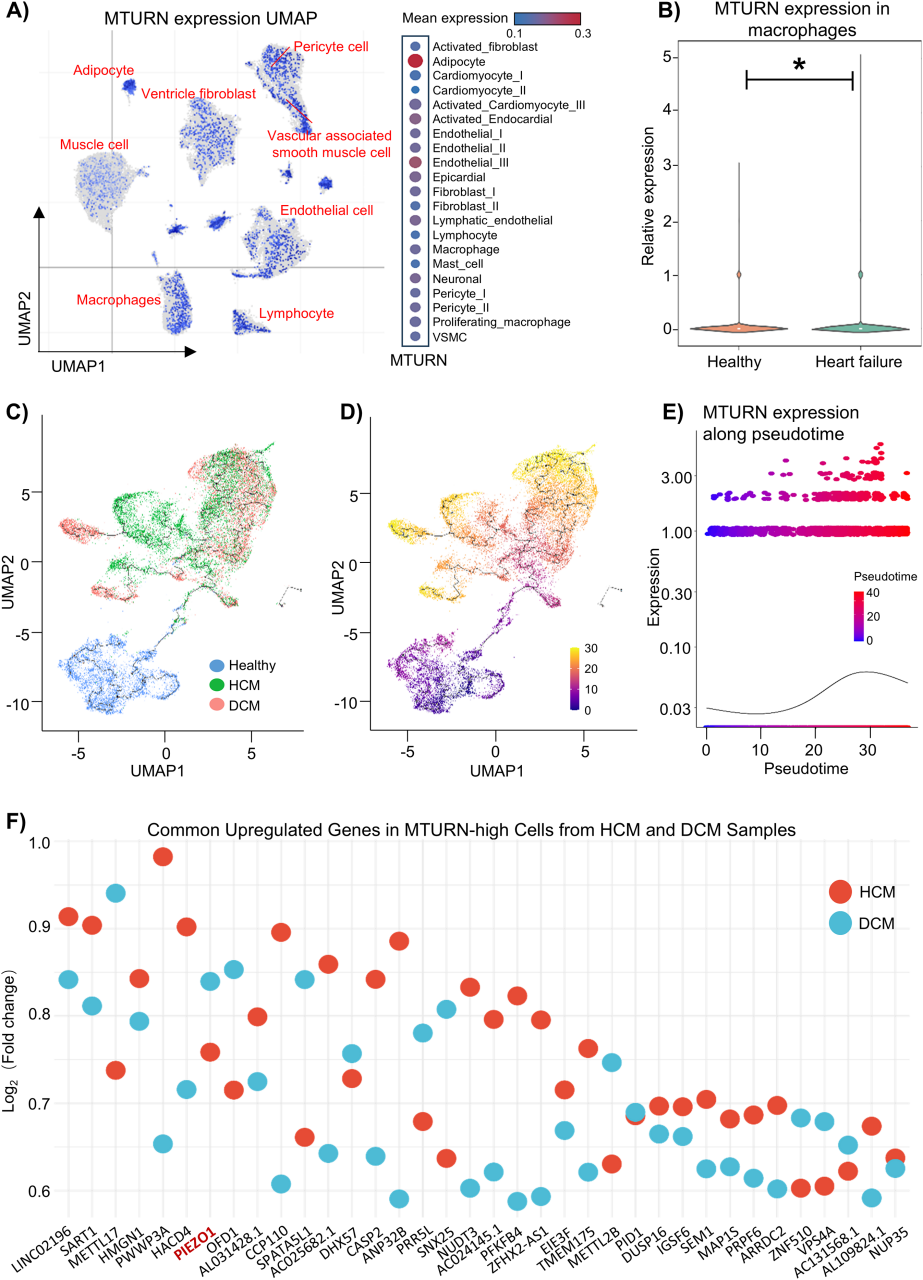
scRNA-seq analysis of MTURN in HCM, DCM patients, and healthy donors from SCP1303 project. **(A)** Dot plot showing the expression distribution of MTURN across major cardiac cell types derived from scRNA-seq data, with dot size representing the proportion of MTURN-expressing cells and color intensity indicating average expression levels. **(B)** Relative expression of MTURN in cardiac macrophages. (HF group *vs*. Healthy group; *p < 0.05). Trajectory analysis of macrophages based on **(C)** sample groups and **(D)** pseudotime. **(E)** Pseudotime expression plot of MTURN. **(F)** Heatmap showing genes co-expressed with MTURN in macrophages from HCM and DCM samples, identified by differential expression analysis between MTURN-high and MTURN-low subsets (|log2FC| ≥ 0.585, *p* < 0.05).

To address the potential role of MTURN in macrophages during the development of heart failure, we selected a scRNA-seq dataset (SCP1303 project from failing human hearts with DCM and HCM) for analysis and UMAP visualization revealed the apparent enrichment in adipocytes, endothelial cells, and macrophages. Further dot-plot analysis results showed that higher expression levels of MTURN were exhibited in adipocytes, followed by endothelial cells, and macrophages, within human hearts (**Figure 6A**, **Supplementary Table 6**). More interestingly, the expression levels of MTURN were increased by 2.1-fold in cardiac macrophages only, but not in either adipocytes or endothelial cells within failing hearts, compared to healthy donors (**Figure 6B**). To explore the dynamic regulation of MTURN during macrophage state transitions, we performed pseudotime trajectory analysis. Cells were ordered along an inferred temporal axis and revealed progressive increase in MTURN expression during macrophage differentiation and activation, followed by stabilization at later pseudotime stages (**Figures 6C-E**). Next, we separated the MTURN-expressing macrophages into high and low expression groups and performed parallel differential expression analysis in HCM and DCM samples (**Figure 6F**, **Supplementary Tables 7**, **8**). This analysis identified several immunity related genes, such as PIEZO1, IGSF6, and EIF3, that were consistently upregulated in MTURN-high macrophages across both disease conditions. Given the prominent enrichment of PIEZO1 in MTURN-high macrophages, we further investigated the dynamic behavior of PIEZO1 at the single-cell level. Pseudotime trajectory analysis revealed that PIEZO1 exhibited a clear pseudotime-dependent expression pattern, characterized by early induction during macrophage state transitions, preceding the upregulation of MTURN, followed by a gradual decline at later stages (**Supplementary Figure S2A**). Although PIEZO1 and MTURN displayed partially distinct temporal dynamics, both genes showed coordinated downregulation toward the terminal pseudotime states, suggesting their involvement in stage-dependent macrophage activation programs rather than terminal differentiation. In addition, macrophages were separated into PIEZO1-high and PIEZO1-low subsets, and comparative analysis demonstrated that MTURN expression was significantly higher in PIEZO1-high group than in PIEZO1-low group (**Supplementary Figure S2B**). Together, these findings suggest a close functional association between PIEZO1 and MTURN during macrophage activation and immune training in heart failure.

### 3.7 MTURN is correlated with heart failure and upregulated by trained immunity in macrophages

**Figure 7:**
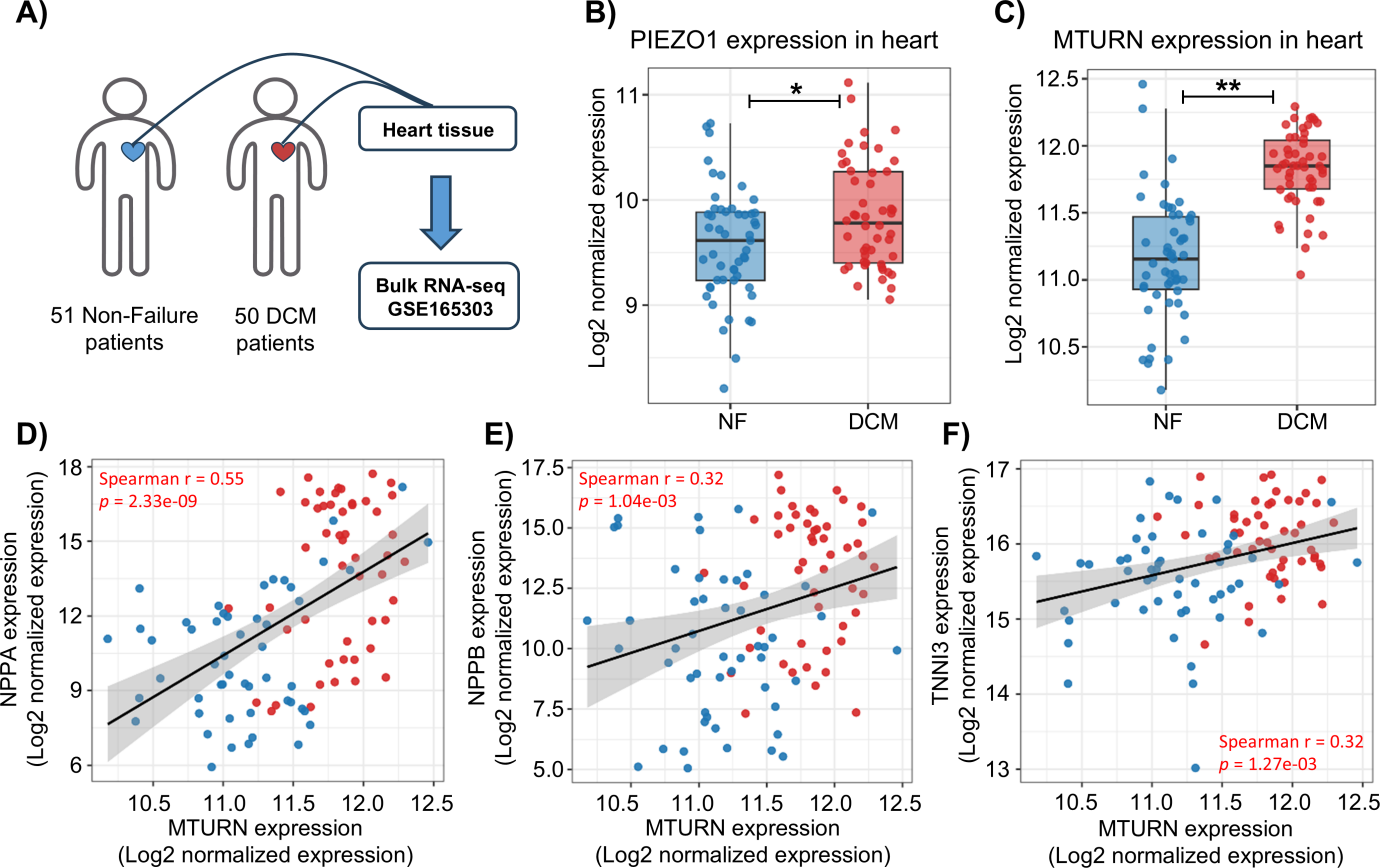
Validation of MTURN and PIEZO1 expression in human heart failure samples. **(A)** Schematic overview of the study design showing bulk RNA-seq analysis of heart tissues from non-failure (NF, n = 51) and dilated cardiomyopathy (DCM, n = 50) patients (GSE165303). The normalized levels of **(B)** PIEZO1 and **(C)** MTURN in heart tissue from NF and DCM groups. Correlation analysis between MTURN and heart failure-related genes **(D)** NPPA, **(E)** NPPB and **(F)** TNNI3. Data are presented as log_2_ normalized expression values; significance was determined by Spearman correlation. *p < 0.05, **p < 0.01.

**Figure 8:**
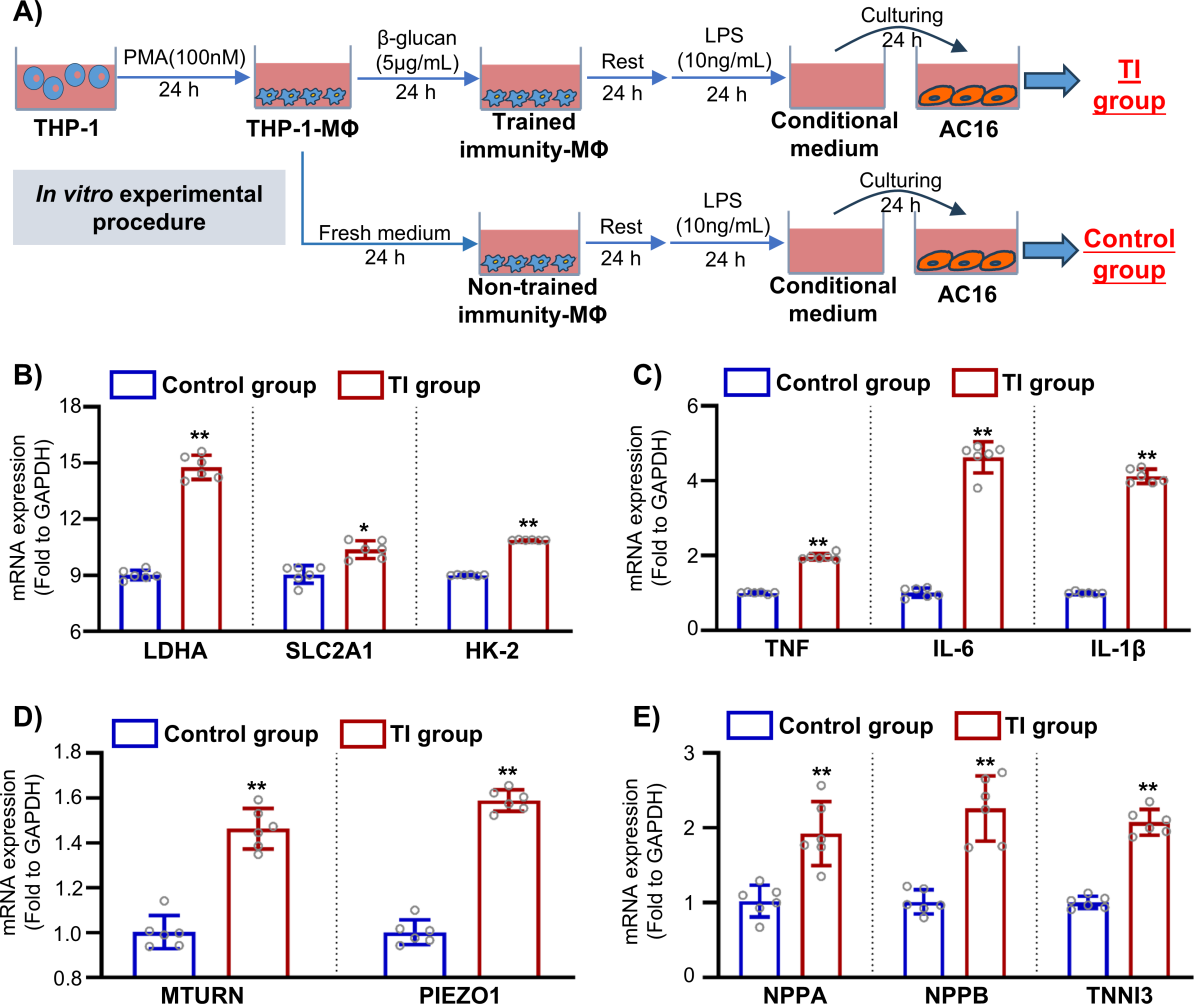
Validation of trained immunity and its paracrine effects on cardiomyocytes. **(A)** Schematic overview of the trained immunity macrophage model and conditioned-medium (CM) treatment of cardiomyocytes. THP-1 monocytes were differentiated into macrophages with PMA (100 nM, 24 h) and trained with β-glucan (5 μg/mL, 24 h), followed by a 24 h rest in complete medium. Cells were then restimulated with LPS (10 ng/mL, 24 h), and the resulting supernatant was collected as trained conditioned medium and applied to AC16 cardiomyocytes (24 h). Control CM was generated in parallel from non-trained THP-1-derived macrophages restimulated with LPS (10 ng/mL, 24 h) and applied to AC16 cells for 24 h. The relative expression of **(B)** glycolytic genes (LDHA, SLC2A1, HK2), **(C)** pro-inflammatory cytokines (TNF, IL-6, IL-1β), **(D)** MTURN and PIEZO1 in trained macrophages, and **(E)** heart failure markers (NPPA, NPPB, TNNI3) in AC16 cells treated with trained-immunity-derived conditioned medium from control or trained macrophages. Data are presented as mean ± SD; *p < 0.05, **p < 0.01 *vs.* control.

To validate our bioinformatics findings, we first revisited our previous bulk RNA-seq dataset (GSE165303) comprising human cardiac samples from healthy controls and patients with DCM (**Figure 7A**). Consistent with our computational analysis, we observed that the expressions of PIEZO1 and MTURN were significantly increased in the DCM group (**Figures 7B, C**). We next examined whether MTURN expression was associated with established heart failure markers at the tissue level. Spearman correlation analysis revealed that MTURN expression was positively correlated with the heart-failure biomarkers NPPA (*r* = 0.55; *p* = 2.33 × 10^-9^), NPPB (*r* = 0.32; *p* = 1.04 × 10^-3^), and TNNI3 (*r* = 0.32; *p* = 1.27 × 10^-3^) (**Figures 7D-F**). In contrast, direct correlation analysis between PIEZO1 and MTURN expression in bulk cardiac tissue did not reveal a significant linear association (Spearman r = 0.07, p = 0.499; as shown in **Supplementary Figure S3**). These results suggest that, although PIEZO1 and MTURN are concurrently upregulated in DCM, their expression levels are not tightly coupled at the whole-tissue level. To further investigate their regulation in a cell-type-relevant context, we established an *in vitro* trained immunity model using human THP-1-derived macrophages and AC16 cardiomyocytes (**Figure 8A**). In trained immunity THP-1-derived macrophages, the mRNA levels of glycolysis-related genes (LDHA, SLC2A1, and HK2) were significantly increased (**Figure 8B**). Meanwhile, the expression of inflammation-related genes (TNF, IL-6, and IL-1β) was also markedly upregulated (**Figure 8C**), indicating successful induction of trained immunity. Notably, trained immunity further upregulated MTURN and PIEZO1 expression in THP-1-derived macrophages (**Figure 8D**), indicating that these two genes are co-responsive under macrophage trained immunity conditions. Finally, conditioned medium collected from these macrophages markedly increased the expression of heart-failure markers (NPPA, NPPB, and TNNI3) in AC16 cardiomyocytes (**Figure 8E**). Collectively, these results validate our bioinformatic analysis and suggest that trained immunity-associated macrophage activation is accompanied by coordinated upregulation of MTURN and PIEZO1. While their expression is not directly correlated at the bulk tissue level, their concordant induction in macrophages supports a potential functional convergence in the context of heart failure-associated immune reprogramming.

## 4 Discussion

**Figure 9:**
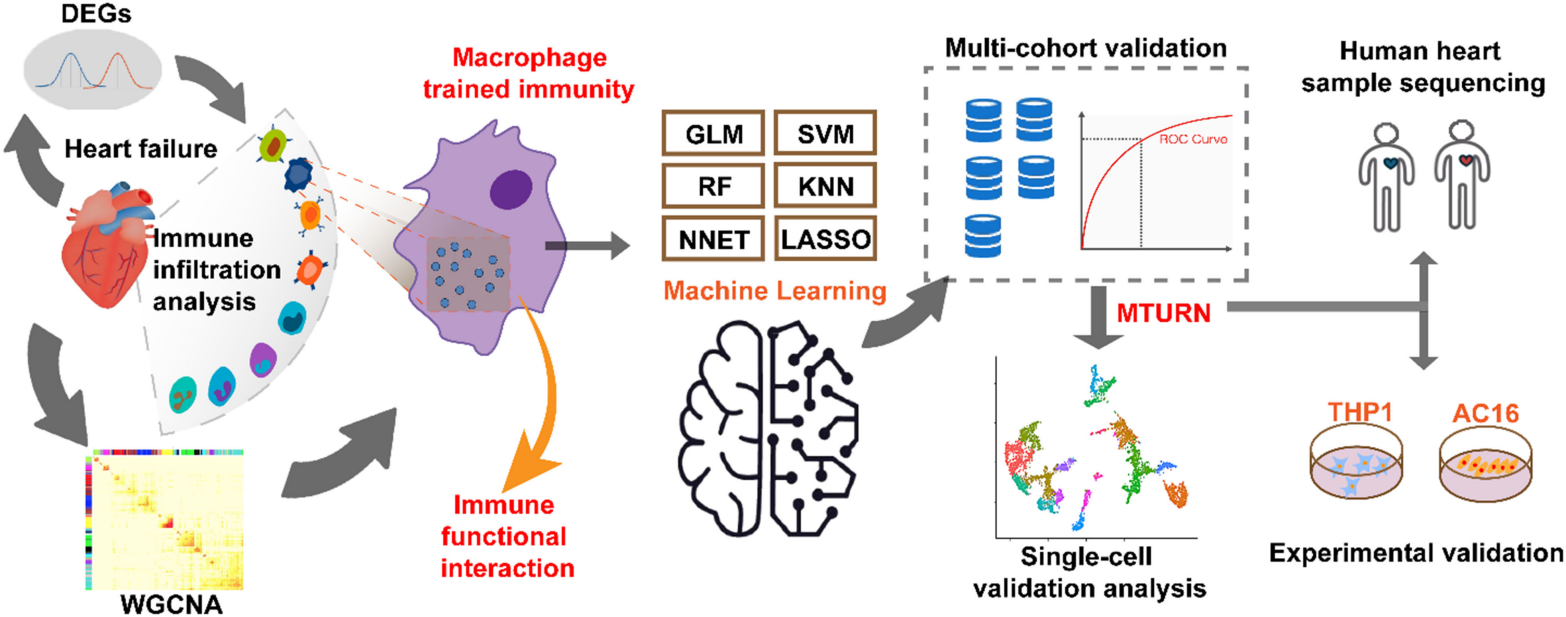
Five heart failure transcriptomic datasets were integrated with a macrophage-trained immunity model to identify immune-related biomarkers. Through DEGs analysis, WGCNA, CIBERSORT, and six machine learning algorithms, hub genes were prioritized with MTURN emerging as the top candidate. Its potential was further validated by scRNA-seq analysis, which confirmed MTURN enrichment in cardiac macrophages. Finally, MTURN expression was validated using previously published heart failure transcriptomic data and *in vitro* experiments.

HF remains one of the leading causes of disability and mortality worldwide, characterized by a high rate of recurrence and hospital readmission. Notably, HF exhibits marked clinical heterogeneity, encompassing distinct subtypes such as HF with preserved ejection fraction (HFpEF) and HF with reduced ejection fraction (HFrEF), for which standard therapies are only partially effective. Despite progress in clinical management and basic research, there remains a lack of reliable and effective biomarkers for both diagnosis and prognosis. In this study, we aimed to identify immune training-related markers in macrophages through functional enrichment analysis of DEGs from HF datasets. By integrating a curated set of trained immunity-related genes, we identified overlapping genes associated with both HF and immune training. We employed multiple machine learning algorithms to prioritize candidate genes and validated the findings across four independent HF cohorts. Among the identified candidates, MTURN demonstrated superior predictive performance. Finally, leveraging scRNA-seq data and validating in separated cohort and *In vitro* experiments, we investigated the association between MTURN expression and disease progression, and identified potentially relevant regulatory genes within the cardiac immune microenvironment (**Figure 9**).

Our enrichment analysis of HF datasets revealed significant activation of multiple pathways associated with innate immune responses (**Figure 1**). These findings prompted us to focus our investigation on functions related to innate immune responses. Notably, immune infiltration analysis revealed a substantial reduction in M2 macrophages within the HF group, which warranted further attention (**Figure 2C**). Importantly, this reduction in M2-like macrophage signatures is not confined to our dataset, as similar trends have been observed across independent heart failure transcriptomic studies (34–37). These findings suggest that impaired or insufficient reparative macrophage responses may coexist with sustained innate immune activation in chronic heart failure, rather than reflecting a contradiction to stage-specific reparative macrophage expansion. To explore this further, we performed correlation analyses between immune subfunctions and gene expression changes using public datasets, revealing a strong and significant association with trained immunity (**Figure 2D**). Previous studies, including that of Yao et al. (45), have suggested that innate immune cells such as macrophages can acquire long-term memory-like states following activation by effector T cells. Interestingly, our data indicated that monocyte differentiation was skewed toward the M1 macrophage phenotype (**Figures 2F, G**). However, in this study, macrophage polarization-related metrics were primarily used as phenotypic indicators of immune reprogramming associated with trained immunity, rather than as a focus on classical polarization signaling pathways. Mechanistically, previous work has shown that trained macrophages undergo a metabolic shift toward aerobic glycolysis and are often active during the early phase of disease. However, M1 macrophages have also been demonstrated to possess the ability to mount sustained responses to microbial stimuli and undergo long-term functional reprogramming, a hallmark of trained immunity (46, 47). In the context of atherosclerosis, non-microbial stimuli such as oxidized LDL and lipoproteins have also been shown to trigger trained immunity in macrophages. While this immune reprogramming may be beneficial under specific conditions, chronic stimulation has been shown to exacerbate atherosclerotic progression (48). Given that heart failure is a chronic condition, it is plausible that a similar mechanism is involved. Notably, previous studies have shown that hematopoietic stem cells (HSCs) isolated from HF mice exhibit a distinct “innate memory” phenotype. When transplanted into healthy mice, these HSCs tended to give rise to pro-inflammatory macrophages, contributing to the progression of HF and multiple comorbidities (49). Integrating these findings, macrophage-mediated trained immunity may contribute to the immunopathogenesis of heart failure, highlighting the need for further in-depth investigation.

To explore the molecular basis of trained immunity in macrophages, we selected a gene set derived from macrophages stimulated with a defined combination of 0.5 ng/mL Al(OH)_3_, 25 ng/mL PHAD, and 0.25 µg/mL Mannan (**Figure 3A**). More broadly, immune training strategies based on Toll-like receptor (TLR) agonists have been shown to induce broader and more controlled immune reprogramming (50). In this regard, PHAD, a low-toxicity synthetic TLR4 agonist, in combination with Al(OH)_3_, not only activates inflammasome pathways but also provides a milder and more physiologically relevant inflammatory stimulus. Meanwhile, Mannan, a Dectin-2 ligand, complements this by promoting M1-like macrophage polarization, aligning with the functional phenotype observed in chronic heart failure. Importantly, this composite stimulus is capable of initiating metabolic and epigenetic reprogramming associated with trained immunity (51, 52). Therefore, this approach represents a β-glucan-like strategy for establishing a stable model of innate immune training, which is well suited for investigating chronic inflammatory diseases such as heart failure. Using transcriptomic data from this model, we identified seven candidate genes—PTGDS, TNFRSF12A, MLLT11, RND3, MTURN, AQP3, and COL23A1—that may serve as key molecular mediators of macrophage immune training in the context of heart failure (**Figure 3I**).

In the complex landscape of molecular identification and mechanistic hypothesis generation, the application of multiple machine learning algorithms has become an essential tool for navigating the “deep waters” of high-dimensional biological data. In the present study, we employed six distinct machine learning algorithms (RF, SVM, GLM, LASSO, KNN, and NNET) (**Figure 4**), aiming to capture nonlinear patterns and extract robust molecular features from a limited number of genes (53, 54). However, based on residual distributions and average variable importance, it remained challenging to determine which algorithm was superior for small-scale gene prioritization. To address this, we applied PCA to compress and integrate model-derived importance scores across algorithms (**Figure 4E**). This dimensionality reduction approach enabled us to identify three consistently top-ranked genes: PTGDS, TNFRSF12A, and MTURN. Among them, MTURN demonstrated the most stable and predictive performance across four independent heart failure datasets included in this study (**Figure 5**). Notably, MTURN expression was consistently elevated in heart failure patients, making this finding particularly compelling. MTURN, also known as C7orf41 or Maturin, was initially characterized by its role in neurogenesis during vertebrate development. Meanwhile, previous studies showed that MTURN synergizes with PAK3 while also exhibiting independent functions in promoting the differentiation of primitive neurons (55). Beyond developmental biology, MTURN has emerged as a potential biomarker in lung cancer, where its diagnostic and predictive utility has been validated across multiple clinical cohorts (56–58). Interestingly, more recent studies have revealed that MTURN can be upregulated in both murine models and human cell lines in response to inflammatory stimuli such as LPS. This upregulation appears to exert an anti-inflammatory effect by suppressing NF-κB signaling activation (59, 60). Earlier work also suggested that MTURN may activate ERK and JNK phosphorylation while inhibiting NF-κB activity, contributing to megakaryocyte differentiation and participating in TPA-induced leukemogenesis. Given that both megakaryocytes and macrophages originate from common myeloid progenitors (CMPs), the possibility of functional or regulatory crosstalk between these cell types remains intriguing, though currently undefined (61). Moreover, MTURN has been shown to regulate keratinocyte proliferation and differentiation in the skin—a primary barrier of the innate immune system—through modulation of IKKα signaling (62). Collectively, these findings indicate that MTURN may possess unrecognized immunomodulatory properties. However, its specific relationship with defined immune cell populations, including macrophages, remains to be fully elucidated. Future research is warranted to clarify its role in innate immune regulation and trained immunity within the context of heart failure and beyond.

To further define the cellular context and dynamic relevance of MTURN in the failing heart, we next interrogated single-cell transcriptomic data. scRNA-seq (SCP1303 project from failing human hearts with DCM and HCM) analysis revealed that MTURN is broadly expressed across multiple cardiac cell types, with notably high expression in immune-related populations (**Figure 6A**). Furthermore, pseudotime trajectory analysis confirmed a strong association between MTURN expression and the progression of heart failure (**Figure 6E**). Moreover, MTURN exhibits strong co-expression with PIEZO1 (**Figure 6F**, **Supplementary Figure S2B**), a widely distributed mechanosensitive ion channel known to transduce diverse mechanical stimuli into intracellular electrochemical signals for downstream molecular signaling (63). In cardiovascular diseases (CVDs), PIEZO1 has been increasingly implicated due to its roles in modulating inflammation and maintaining mitochondrial homeostasis, and it is now considered a promising candidate for novel CVD therapies (64). In recent years, emerging evidence has demonstrated a substantial connection between PIEZO1 and innate immunity. Under cyclical hydrostatic pressure, PIEZO1 activation induces macrophage expression of multiple pro-inflammatory genes such as CXCL10 and PTGS2, along with the secretion of EDN1. This signaling cascade exerts dual effects: while it enhances antibacterial defense, it also exacerbates the progression of pathological fibrosis in organs such as the heart, kidneys, and liver (64–68). These findings closely parallel the previously reported deleterious effects of trained immunity in chronic fibrotic diseases. In addition, PIEZO1 and TLR4 exhibit strong synergistic signaling activity. Their cooperative interaction activates the CaMKII-Mst1/2-Rac axis, thereby amplifying the antibacterial functions of macrophages (69). This specific synergism has also been directly associated with tissue damage in sepsis-induced intestinal and cardiac injury models (70, 71). Consistent with the scRNA-seq observations, our previous bulk RNA-seq cohort (GSE165303) showed significant upregulation of both MTURN and PIEZO1 in DCM heart tissue (**Figures 7A-C**), and MTURN was positively correlated with established HF biomarkers (NPPA, NPPB, and TNNI3) (**Figures 7D-F**). Notably, correlation analysis indicated that MTURN and PIEZO1 were not significantly associated at the whole-tissue level (**Supplementary Figure S3**). This could be ascribed to cellular heterogeneity and distinct regulatory inputs across cardiac compartments. Furthermore, we established a trained-immunity model in THP-1-derived macrophages using a β-glucan-based protocol (**Figure 8**), independent of the Al(OH)_3_/PHAD/mannan paradigm used to generate the transcriptomic signature. Interestingly, the expressions of MTURN and PIEZO1 were concordantly upregulated in THP-1 macrophages upon stimuli with immune training. In addition, conditioned medium from trained macrophages markedly increased the expression of heart failure-related genes in cardiomyocytes, supporting a paracrine contribution of trained macrophages to cardiomyocyte dysfunction. Collectively, these findings suggest that the coordinated upregulation of MTURN and PIEZO1 may be linked to sustained trained-immunity programs in heart failure, potentially exacerbating chronic inflammatory-induced pathological cardiac remodeling.

## 5 Conclusion

Based on our analyses, MTURN may serve as a key target gene involved in macrophage-mediated trained immunity during the progression of heart failure. Its synergistic interaction with PIEZO1 potentially underpins the immune training-related mechanisms driving disease exacerbation. Although the *in vivo* functional relevance of this interaction remains to be elucidated, our findings could provide a foundation for future studies aimed at defining the co-regulatory roles of MTURN and PIEZO1 in the progress of heart failure.

## Data Availability

The datasets presented in this study can be found in online repositories. The names of the repository/repositories and accession number(s) can be found in the article/**Supplementary Material**.
