# Supplementary material for: Identification of MTURN as a trained immunity-related biomarker for heart failure via integrative transcriptomic machine learning analysis and experimental validation
Source: Front Immunol. 2026 Feb 18;17:1739660. doi: 10.3389/fimmu.2026.1739660 (PMC12957145; doi:10.3389/fimmu.2026.1739660)
Supplement: Supplementary file 9 [file DataSheet9.docx]

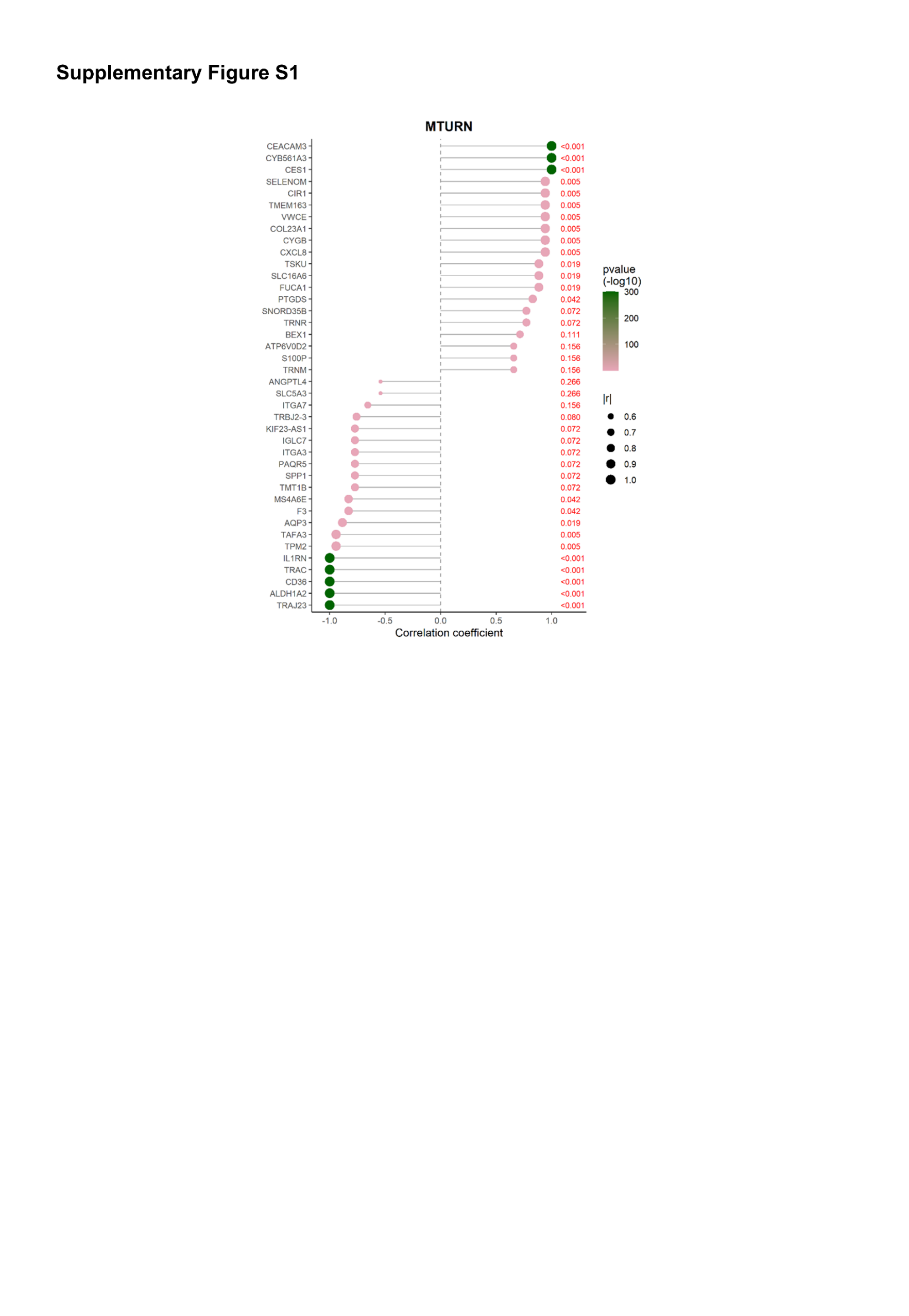


**Supplementary Figure S1.** Correlation analysis between MTURN expression and trained immunity-related genes in the GSE235897 dataset. Bulk RNA-seq data from GSE235897, in which human monocytes were differentiated into macrophages and subjected to trained immunity induction, were analyzed. Differentially expressed genes associated with macrophage-trained immunity were identified and used for correlation analysis with MTURN expression. Spearman correlation coefficients were calculated across all samples. The dot-lollipop plot shows the correlation coefficient for each gene, with genes ordered by correlation direction and magnitude. The dashed vertical line indicates zero correlation. Dot size represents the absolute correlation coefficient (|r|), and dot color denotes statistical significance expressed as -log10(*p* value). Individual p values are shown on the right side of the plot.


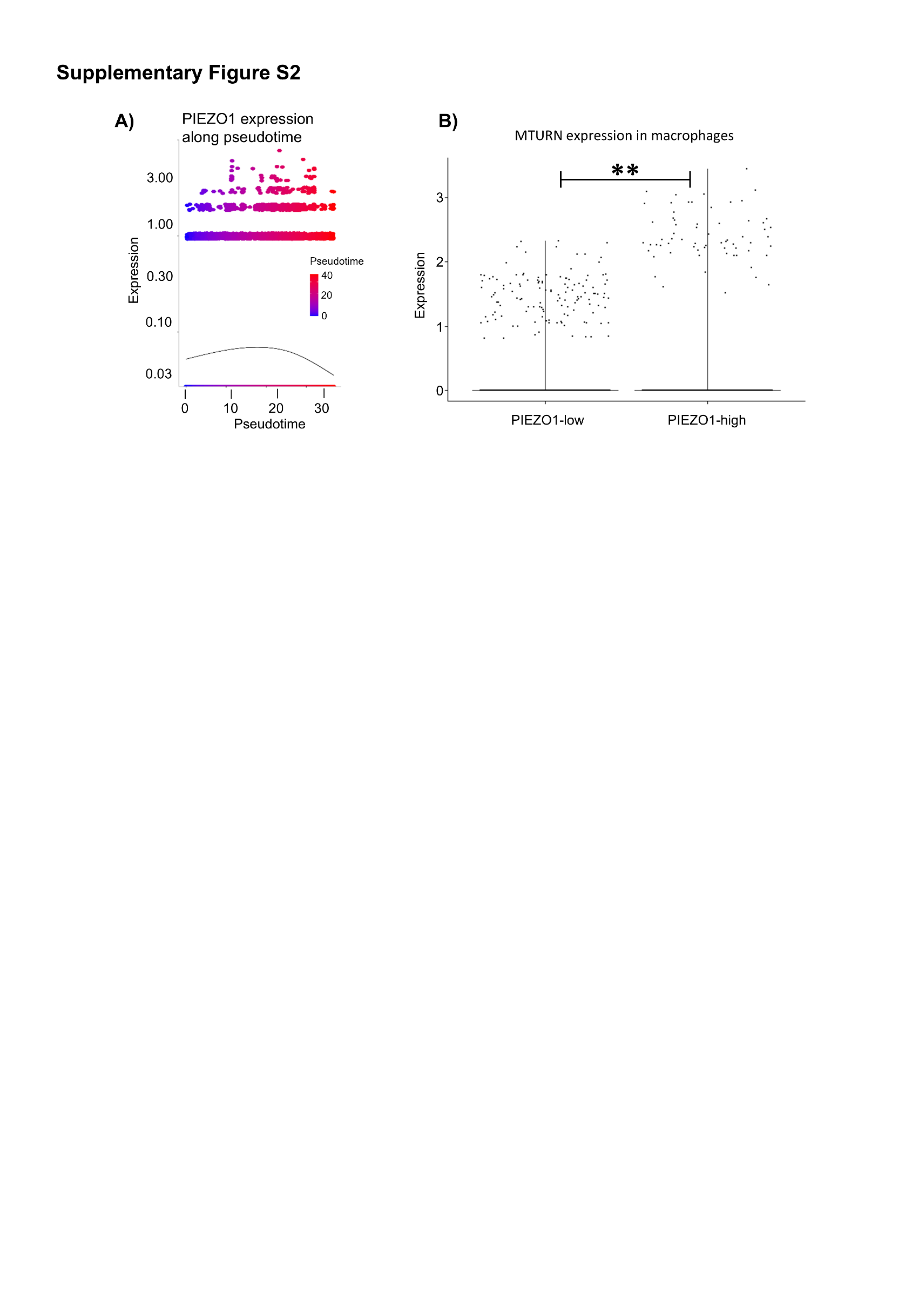


**Supplementary Figure S2**. The analysis of single-cell RNA-seq dataset from SCP1303 project reveals a positive correlation between the expression of PIEZO1 and MTURN in cardiac macrophages. (A) PIEZO1 expression in cardiac macrophages along pseudotime. Trajectory inference was performed in cardiac macrophages to generate pseudotime ordering. Each dot represents a single cell, colored by pseudotime value. The grey curve indicates the smoothed trend of PIEZO1 expression across pseudotime, showing the increased expression at the early stage, followed by a gradual decline at later stage. (B) MTURN expression pattern in cardiac macrophages is stratified by PIEZO1 level. Macrophages were divided into PIEZO1-high and PIEZO1-low subsets (top and bottom 40% of PIEZO1 expression, with intermediate cells excluded). Each dot represents a single cell. MTURN expression was significantly higher in the PIEZO1-high subset compared with the PIEZO1-low subset (*p* < 0.01, Wilcoxon rank-sum test).


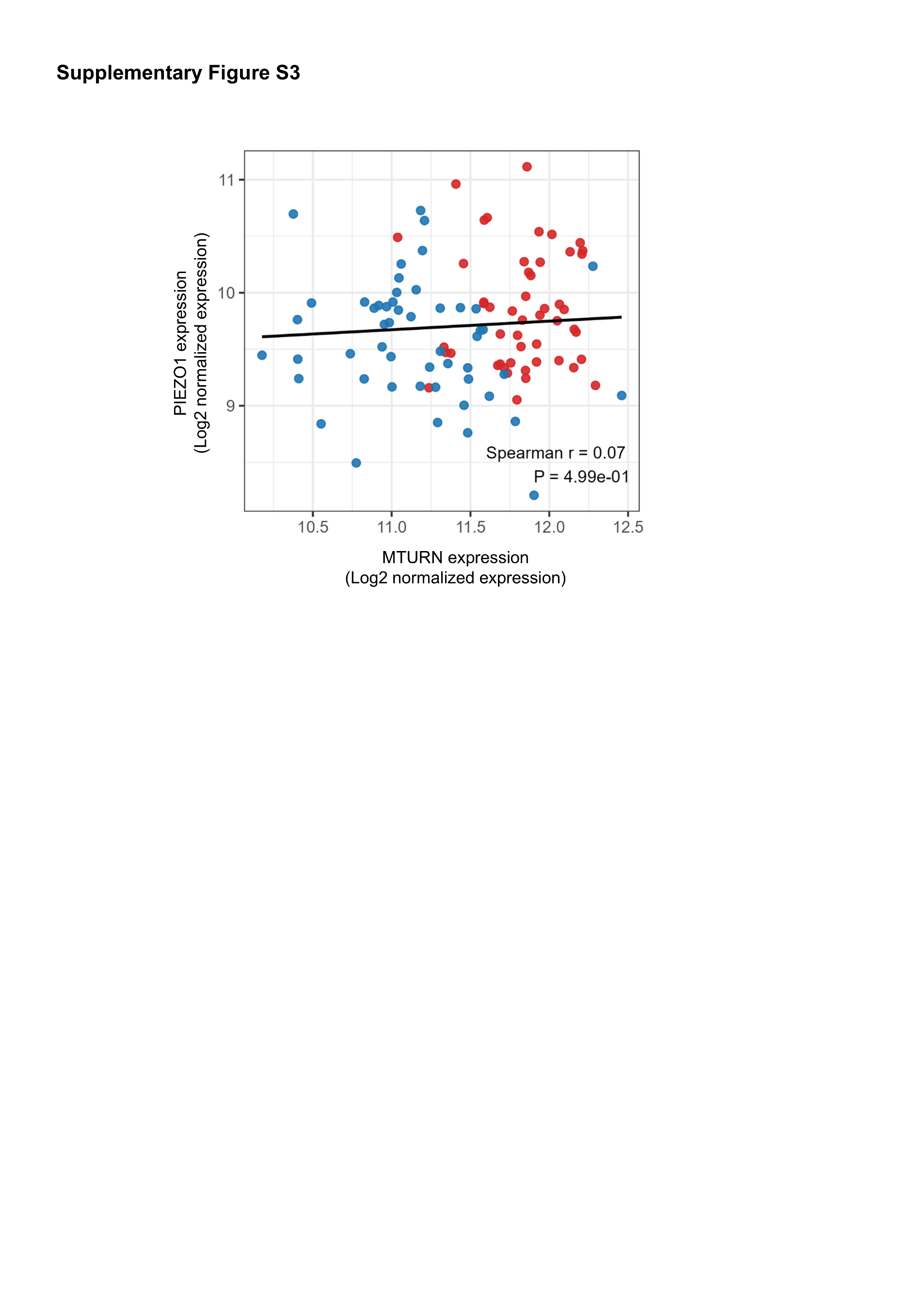


**Supplementary Figure S3**. Correlation analysis between MTURN and PIEZO1 in human heart tissues. Scatter plot showing the relationship between MTURN and PIEZO1 expression in heart tissue samples from the NF (blue, n = 51) and DCM (red, n = 50) groups in GSE165303. Expression values are shown as log₂-normalized expression. The black line indicates the fitted trend. Correlation significance was determined by Spearman correlation (Spearman r = 0.07, P = 4.99 × 10⁻¹).
