# Supplementary material for: Identification of MTURN as a trained immunity-related biomarker for heart failure via integrative transcriptomic machine learning analysis and experimental validation
Source: Front Immunol. 2026 Feb 18;17:1739660. doi: 10.3389/fimmu.2026.1739660 (PMC12957145; doi:10.3389/fimmu.2026.1739660)
Supplement: Supplementary file 10 [file DataSheet10.docx]

**Supplementary Methods**

1 Dataset collection

GSE135055 (<https://www.ncbi.nlm.nih.gov/geo/query/acc.cgi?acc=GSE135055>) contains bulk RNA-seq profiles of left ventricular (LV) myocardial tissues obtained from 21 patients with heart failure (HF) and 9 non-failing healthy donors. Sequencing was performed on the Illumina HiSeq 2500 platform. In this study, this dataset served as the primary discovery cohort and was used for differential gene expression (DEGs) analysis, immune cell deconvolution using CIBERSORT, weighted gene co-expression network analysis (WGCNA), and training of machine-learning models.

GSE198945 (<https://www.ncbi.nlm.nih.gov/geo/query/acc.cgi?acc=GSE198945>), includes bulk RNA-seq data from left ventricular tissues of 20 HF patients and 20 healthy controls, generated using the Illumina NovaSeq 6000 platform. This dataset was used as an independent validation cohort to confirm expression differences of MTURN and other candidate genes and to evaluate their diagnostic performance.

GSE48166 (<https://www.ncbi.nlm.nih.gov/geo/query/acc.cgi?acc=GSE48166>), comprises bulk transcriptomic profiles from 15 ischemic cardiomyopathy (ICM) hearts and 15 non-failing (NF) control hearts, collected at the time of heart transplantation. Sequencing was performed using Illumina Genome Analyzer II and SOLiD platforms. This cohort was included to assess the robustness of candidate biomarkers across platforms and in ischemic cardiomyopathy–specific heart failure.

GSE116250 (<https://www.ncbi.nlm.nih.gov/geo/query/acc.cgi?acc=GSE116250>), contains 64 human left ventricular samples, including 14 NF, 37 dilated cardiomyopathy (DCM), and 13 ICM samples. Poly(A)-enriched RNA sequencing was conducted on the Illumina HiSeq 2500 platform. This dataset was used for cross-cohort validation and to examine the stability of MTURN expression across different heart failure etiologies.

GSE203160 (<https://www.ncbi.nlm.nih.gov/geo/query/acc.cgi?acc=GSE203160>), includes bulk RNA-seq data from human end-stage ischemic heart disease (IHD) left ventricular tissues and controls, with a total of 15 samples sequenced on the Illumina NextSeq 500 platform. Although the GEO series lists 15 samples without explicit group annotation, this cohort is commonly described in downstream analyses as comprising 8 ICM/IHD cases and 7 controls. In this study, GSE203160 was used as an additional independent cohort for validating candidate gene expression patterns and ROC performance.

GSE235897 (<https://www.ncbi.nlm.nih.gov/geo/query/acc.cgi?acc=GSE235897>), provides bulk RNA-seq data from human peripheral blood mononuclear cell derived myeloid cells subjected to an adjuvant-based trained-immunity stimulation (A + M + MA). A total of 12 samples were sequenced on the Illumina NovaSeq 6000 platform, including monocytes (n = 3), naïve macrophages (n = 3), trained macrophages at day 3 post-stimulation (MMA-3d, n = 3), and trained macrophages at day 6 post-stimulation (MMA-6d, n = 3). Each condition represents an independent biological replicate. This dataset was used to derive a transcriptional signature associated with macrophage trained immunity and to perform correlation analyses centered on MTURN and trained immunity-related genes.

Single-cell transcriptomic data were obtained from the SCP1303 project ([https://singlecell. broadinstitute.org/single_cell/study/SCP1303/single-nuclei-profiling-of-human-dilated-and-hypertrophic-cardiomyopathy](https://singlecell.broadinstitute.org/single_cell/study/SCP1303/single-nuclei-profiling-of-human-dilated-and-hypertrophic-cardiomyopathy)). This dataset corresponds to the published single-nucleus RNA-seq (snRNA-seq) study “Single-nuclei profiling of human dilated and hypertrophic cardiomyopathy” and includes approximately 600,000 nuclei isolated from human left ventricular tissues, comprising 11 DCM hearts, 15 hypertrophic cardiomyopathy (HCM) hearts, and 16 non-failing donor hearts. In the present study, SCP1303 was used to assess the cellular distribution of MTURN expression across major cardiac cell populations (UMAP and dot plot analyses), to evaluate macrophage-specific enrichment, and to perform macrophage pseudotime and trajectory inference analyses.

2 Immune infiltration and functional analysis

To evaluate the immune landscape across samples, we implemented a gene set-based scoring strategy using normalized FPKM values. A curated panel of ten immune-related functional pathways was selected based on prior immunology and immunotherapy studies, covering both innate and adaptive immune processes. These included: Trained Immunity, Type I Interferon Response, Antigen Presentation, Macrophage Polarization, Dendritic Cell Activation, T Cell Activation, B Cell Activation, Natural Killer Cell Activation, Cytokine Production, Inflammatory Response. These pathways were chosen for their relevance to host immune regulation and their reported dysregulation in chronic inflammatory diseases, including heart failure (1-7). Notably, trained immunity was highlighted for its role in innate immune memory and metabolic reprogramming. For each sample, we quantified the activity of each immune pathway via a Z-score-based enrichment approach:

- For a given immune pathway *i*, we defined a gene set G*ᵢ* containing known marker genes.
- The expression values (FPKM) of genes in G*ᵢ* were Z-score normalized across samples.
- The mean Z-score of all genes in G*ᵢ* was computed for each sample, yielding a pathway-specific score.

This process resulted in a 10 × N immune score matrix, where rows represent immune functions and columns correspond to samples. Formula:

$$Score_{i,j}=\frac{1}{\left| G_{i} \right|}\sum_{g\in G_{i}} Z_{g,j}$$

**References:**

1. Ochando J, Mulder WJM, Madsen JC, Netea MG, Duivenvoorden R. Trained immunity - basic concepts and contributions to immunopathology. *Nat Rev Nephrol*. (2023) 19(1):23-37. doi: 10.1038/s41581-022-00633-5.
2. Mazewski C, Perez RE, Fish EN, Platanias LC. Type I Interferon (IFN)-Regulated Activation of Canonical and Non-Canonical Signaling Pathways. *Front Immunol*. (2020) 23;11:606456. doi: 10.3389/fimmu.2020.606456.
3. Muntjewerff EM, Meesters LD, van den Bogaart G. Antigen Cross-Presentation by Macrophages. *Front Immunol*. (2020) 8;11:1276. doi: 10.3389/fimmu.2020.01276.
4. Harvey AG, Graves AM, Uppalapati CK, Matthews SM, Rosenberg S, Parent EG, et al. Dendritic cell-natural killer cell cross-talk modulates T cell activation in response to influenza A viral infection. *Front Immunol*. (2022) 22;13:1006998. doi: 10.3389/fimmu.2022.1006998.
5. Netea MG, Joosten LA, Latz E, Mills KH, Natoli G, Stunnenberg HG, et al. Trained immunity: A program of innate immune memory in health and disease. *Science*. (2016) 22;352(6284):aaf1098. doi: 10.1126/science.aaf1098.
6. Cui A, Huang T, Li S, Ma A, Pérez JL, Sander C, et al. Dictionary of immune responses to cytokines at single-cell resolution. *Nature*. (2024) 625(7994):377-384. doi: 10.1038/s41586-023-06816-9.
7. Barreira da Silva R, Münz C. Natural killer cell activation by dendritic cells: balancing inhibitory and activating signals. *Cell Mol Life Sci*. (2011) 68(21):3505-18. doi: 10.1007/s00018-011-0801-8.
